# Impact of the Atomic Structure at the BiVO_4_/TiO_2_ Interface on the Electronic Properties and Performance of BiVO_4_/TiO_2_ Photoanodes

**DOI:** 10.1021/jacs.5c07695

**Published:** 2025-08-14

**Authors:** Dae Han Wi, Kana Ishisone, Zhaoyi Xi, Zifan Ye, Daye Seo, Jiawei Zhan, Xiao Tong, Mingzhao Liu, Giulia Galli, Kyoung-Shin Choi

**Affiliations:** 1 Department of Chemistry, 5228 University of Wisconsin-Madison, Madison, Wisconsin 53706, United States; 2 Department of Chemistry, Chungnam National University, Daejeon 34134, Republic of Korea; 3 Pritzker School of Molecular Engineering, 2462 University of Chicago, Chicago, Illinois 60637, United States; 4 Center for Functional Nanomaterials, 8099 Brookhaven National Laboratory, Upton, New York 11973, United States; 5 Department of Materials Science and Chemical Engineering, Stony Brook University, Stony Brook, New York 11794, United States; 6 Department of Chemistry, 2462 University of Chicago, Chicago, Illinois 60637, United States; 7 Argonne National Laboratory, Lemont, Illinois 60439, United States

## Abstract

In photoelectrochemical cells, semiconductor electrodes are usually interfaced with protection layers to extend their stability. Ideally, the protection layer should not decrease photocurrent generation. Hence, the conduction band minimum (CBM) and valence band maximum (VBM) of the protection layer should appropriately align with those of the underlying semiconductor electrode to facilitate the desired interfacial charge transfer with minimal interfacial recombination. However, predicting interfacial band alignment can be challenging, as it may vary depending on the detailed interfacial atomic structure. Investigating the effect of the atomic structure at the semiconductor/protection layer junction on the band alignment is also challenging as it requires samples with varied interfaces without altering the semiconductor and protection layers. Here, we considered TiO_2_, the most widely used material as a protection layer, interfaced with a BiVO_4_ photoanode, and we fabricated two n-type BiVO_4_(010)/TiO_2_ photoanodes where a thin (∼4 nm) amorphous TiO_2_ layer was deposited by atomic layer deposition (ALD). While the individual BiVO_4_(010) and TiO_2_ layers were identical in these two samples, we modified the interfacial atomic structure at the BiVO_4_/TiO_2_ junction by changing which precursor, Ti or O, was introduced first upon deposition of TiO_2_. By experimentally and computationally investigating the differences in these two samples, we show that the band alignments between BiVO_4_ and TiO_2_ at the interface may not be straightforwardly predicted by the CBM and VBM of bulk BiVO_4_ and TiO_2_ and that interfacial atomic arrangements can have a marked impact on the electronic properties and photoelectrochemical performance of the BiVO_4_(010)/TiO_2_ photoanode.

## Graphical abstract

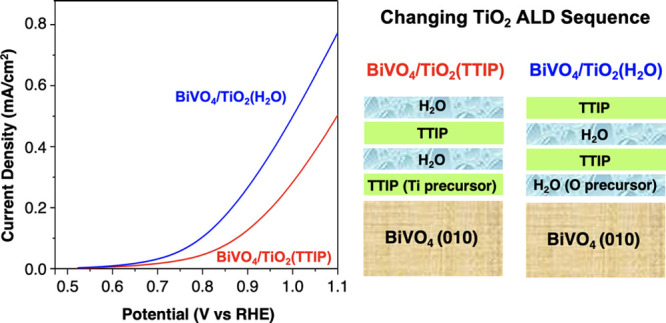

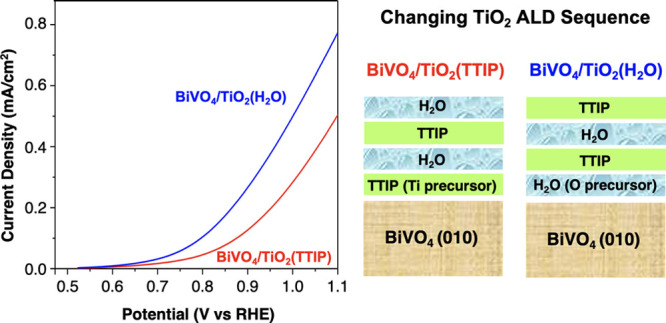

## Introduction

Photoelectrochemical cells (PEC) can drive thermodynamically uphill chemical reactions (e.g., water splitting) with no additional energy input.^1^ This is because semiconductor electrodes used in a PEC (referred to as photocathode or photoanode) can absorb photons from the sun and generate charge carriers (electrons in the conduction band (CB) and holes in the valence band (VB)) at energy levels sufficient to drive the chemical reactions.^2,3^

Since the semiconductor electrodes used in PECs are in direct contact with electrolyte, they should be stable in the electrolyte used in the PEC. If the PEC needs to operate outside the pH range where the semiconductor electrode is stable for various reasons, a protection layer can be used to prevent direct contact between the electrolyte and the semiconductor electrode.^4–8^ The protection layer can also be used to prevent photocorrosion,^9–11^ where the photogenerated electrons and holes reduce or oxidize the semiconductor itself instead of the desired species in the electrolyte. As photocorrosion of the semiconductor involves a change in its composition, placing a chemically and electrochemically inert protection layer between the semiconductor and electrolyte can suppress photocorrosion. When a protection layer is used, the electrons and holes not consumed for photocorrosion should ideally be used for photocurrent generation instead of electron–hole recombination; hence, it is desirable for the protection layer to have favorable band positions to enable the extraction of the charge carriers from the semiconductor and transport them to the electrolyte or a catalyst deposited on top of the protection layer.

The most extensively used material for protecting photoelectrodes is TiO_2_ because TiO_2_ is chemically and electrochemically stable under a wide range of pH and potential conditions.^4–13^ For example, TiO_2_ has been used to suppress photocorrosion of Cu_2_O photocathodes^9,10^ or enable the use of BiVO_4_ photoanodes in basic solutions where BiVO_4_ is not chemically stable.^5^ However, our understanding of semiconductor/TiO_2_ interfaces is still very limited. For example, while TiO_2_ has been used to protect photoanodes like n-type BiVO_4_ and n-type Si, the valence band maximum (VBM) of TiO_2_ is more positive (∼3 V vs SHE) than those of BiVO_4_ (∼2.4 V vs SHE)^5,14,15^ and n-Si (∼0.5 V vs SHE).^7,8^ Thus, the VBM of TiO_2_ should not be able to efficiently accept holes from like n-type BiVO_4_ and n-type Si. However, sizable amounts of anodic photocurrent have been generated from BiVO_4_/TiO_2_ and n-Si/TiO_2_ photoanodes. From these observations, it was believed that the holes from the semiconductor are transferred to TiO_2_ not through the VBM but through the defect levels of TiO_2_.^7,8,14^

In our recent study on a BiVO_4_/FeOOH photoanode where a thin FeOOH layer on BiVO_4_ is used as an oxygen evolution catalyst,^16^ we showed that the band alignment at the BiVO_4_/FeOOH interface is quite different from those expected based on the conduction band minimum (CBM) and VBM of individual bulk BiVO_4_ and FeOOH phases. Also, we showed that the detailed atomic arrangements and bonding nature at the BiVO_4_/FeOOH interface can considerably change the interfacial band alignment even when the same BiVO_4_ and FeOOH layers are paired.

These findings prompted the question of whether the band alignment at the BiVO_4_/TiO_2_ interface is also quite different from those expected from the CBM and VBM of bulk BiVO_4_ and TiO_2_ depending on the interfacial atomic structure. We recognized that there is a lack of understanding of the electronic structure of the interface and the interplay between interfacial atomic structure, bonding, and electronic properties. In this study, to isolate and investigate the effect of the atomic structure at the BiVO_4_/TiO_2_ junction on the electronic properties and performance of BiVO_4_/TiO_2_ photoanodes, we prepared two BiVO_4_/TiO_2_ samples where the BiVO_4_ and TiO_2_ layers are identical but the BiVO_4_/TiO_2_ interface is varied. By experimentally and theoretically comparing these two samples, we demonstrate that the band alignment at the BiVO_4_/TiO_2_ interface cannot be straightforwardly predicted by the CBM and VBM of bulk BiVO_4_ and TiO_2_ and that the band alignment at the BiVO_4_/TiO_2_ junction is sensitive to the detailed interfacial atomic structures even when identical BiVO_4_ and TiO_2_ layers are interfaced.

## Methods

### Experimental Methods

#### Epitaxial BiVO_4_(010) Film Preparation

Pristine BiVO_4_ thin films were fabricated on yttria-stabilized zirconia (YSZ) (100) single-crystal substrates (MTI Corp) via pulsed laser deposition (PLD) using a KrF excimer laser (λ = 248 nm) operating at a repetition rate of 20 Hz with a fluence of 1.8 J/cm^2^ at the target to ensure stoichiometric target-to-film elemental transfer.^17^ The ceramic BiVO_4_ target was prepared by pressing commercial BiVO_4_ powder (Alfa Aesar, 99.9%, ∼200 mesh) into pellets and sintering them at 710 °C for 10 h. Before the deposition of BiVO_4_, a 50 nm thick indium tin oxide (ITO) layer was first deposited on the YSZ substrate at 600 °C under a base pressure of 6 × 10^–7^ Torr, serving as a conductive back contact. Then the BiVO_4_ film was deposited at 675 °C under an oxygen pressure of 20 mTorr. After cooling to room temperature, the samples were unloaded from the PLD chamber. The thickness of the BiVO_4_ film was 140 nm.

#### Atomic Layer Deposition (ALD) of TiO_2_ on BiVO_4_(010)

A TiO_2_ protection layer was deposited on BiVO_4_ by atomic layer deposition (ALD) (Cambridge Nanotech Savannah S100) at 120 °C, using titanium tetraisopropoxide (TTIP, preheated to 80 °C) as the Ti precursor and water vapor (room temperature) as the O precursor, with N_2_ as the carrier gas (590 mTorr, 20 sccm) that flowed constantly. Each ALD cycle was composed of a TTIP half-cycle and a water half-cycle. In the TTIP half-cycle, TTIP vapor was introduced into the chamber for 150 ms, followed by 15 s of N_2_ purging. In the water half-cycle, water vapor was introduced into the deposition chamber for 15 ms, followed by 15 s of N_2_ purging. By changing which half-cycle was used first, two BiVO_4_/TiO_2_ samples were prepared. When the ALD of TiO_2_ was initiated with the TTIP cycle, the resulting sample was named BiVO_4_/TiO_2_(TTIP). When the ALD of TiO_2_ was initiated with the water cycle, the resulting sample was named BiVO_4_/TiO_2_(H_2_O). The TiO_2_ layers on both BiVO_4_/TiO_2_(TTIP) and BiVO_4_/TiO_2_(H_2_O) samples were grown by the same 123 ALD cycles to achieve the same thickness. The 123 cycles were chosen to prepare ∼5 nm thick TiO_2_ layer based on the nominal deposition rate of 0.0407 nm/cycle, which was determined via fitting spectroscopic ellipsometry (J.A. Woollam, M-2000) results from TiO_2_ films deposited on a silicon wafer.^10^ The actual thicknesses of the TiO_2_ layers on BiVO_4_ were 3.54 ± 0.17 nm in BiVO_4_/TiO_2_(TTIP) and 3.75 ± 0.12 nm in BiVO_4_/TiO_2_(H_2_O).

#### Characterization

X-ray diffraction (XRD) patterns of the samples were obtained using an X-ray diffractometer (Bruker D8 Discover) with a Cu Kα (λ = 1.54178 Å) radiation source. UV–vis absorbance spectra of the samples were collected with a UV–vis spectrometer (Agilent Technology, Cary 5000 UV–vis–NIR Spectrophotometer). The ITO/YSZ film was used as a reference to calibrate the background for these measurements. Transmission electron microscopy (TEM) images of the samples were obtained with a field emission transmission electron microscope (FEI Tecnai TF 30) operated at 300 kV. X-ray photoelectron spectroscopy (XPS) spectra were obtained using an X-ray photoelectron spectrometer (Thermo K-Alpha X-ray Photoelectron Spectrometer) with an Al Kα X-ray source. For the calibration of the XPS data, the C 1s peak at 284.8 eV was used as the reference.

#### Photoelectrochemical and Electrochemical Characterization

Photoelectrochemical measurements were carried out in an undivided three-electrode cell using an SP-200 potentiostat (Bio-Logic). BiVO_4_/TiO_2_ was used as the working electrode, Pt was used as the counter electrode, and a double-junction Ag/AgCl (4 M KCl) electrode was used as the reference electrode (RE). The measured potential versus the RE was converted to potential versus the reversible hydrogen electrode (RHE) using the following equation.

$$\begin{array}{c} E\left(\mathrm{vs}\;\mathrm{RHE}\right)=E\left(\mathrm{vs}\;\mathrm{Ag}/\mathrm{AgCl}\right)+E_{\mathrm{Ag}/\mathrm{AgCl}}\left(\mathrm{reference}\right)+0.0591\;V\;\times \mathrm{pH}\\ \left(E_{\mathrm{Ag}/\mathrm{AgCl}}\left(\mathrm{reference}\right)=0.1976\;V\;\mathrm{vs}\;\mathrm{NHE}\;\mathrm{at}\;25{\text{°}}^{\;}C\right) \end{array}$$

An LCS-100 solar simulator (Oriel Instruments) equipped with 100 W Xe lamp, AM 1.5G filter and water IR filter (Newport) was used as a light source. The light intensity was calibrated to 1 sun (100 mW/cm^2^) at the back side of the BiVO_4_/TiO_2_ electrode by an NREL-certified GaAs reference cell (PV Measurements) covered with a 3 mm thick quartz plate (same thickness as the undivided quartz cell used for the photoelectrochemical experiments). The working electrode was oriented so that the light illuminated from the back side, and the Pt counter electrode was oriented perpendicular with respect to the working electrode to eliminate the reflected light by the Pt surface. A borate buffer solution used as the electrolyte was prepared by dissolving 0.5 M boric acid (H_3_BO_3_, Alfa Aesar, 99.8%) and adjusting the pH of the solution to 9.3 with KOH (Sigma-Aldrich, ≥ 85%). Then, 0.4 M sodium sulfite (Na_2_SO_3_, Sigma-Aldrich, ≥ 98.0%) was dissolved in the borate buffer.

Electrochemical impedance spectra were collected at 0.65 and 0.7 V vs RHE using a 10-mV amplitude perturbation in the frequency range of 10,000 to 0.2 Hz under the same conditions used for photocurrent measurement. The potentials of 0.65 and 0.7 V vs RHE were chosen because they are near the photocurrent onsets of the two samples where the difference in photocurrent between the two samples is most pronounced. As discussed below, by comparing Nyquist plots of BiVO_4_, BiVO_4_/TiO_2_(TTIP), and BiVO_4_/TiO_2_(H_2_O), we identified that a semicircle in the low frequency region is from the addition of the TiO_2_ layer. From this low frequency semicircle, we obtained the charge transfer resistance (*R*_CT_) and interfacial charging capacitance (*C*_IT_) of the TiO_2_ layer. Detailed procedures used to obtain these values are provided in the SI.

### Computational Methods

#### Modeling Amorphous TiO_2_ (a-TiO_2_)

In several previous studies, amorphous structural models of TiO_2_ were generated using first-principles molecular dynamics (FPMD) and a “melt-and-quench” procedure.^18,19^ However, such a strategy is computationally demanding and usually limited to relatively small simulation cells and fast quenching rates. Here we prepared amorphous TiO_2_ (a-TiO_2_) samples using a recently developed deep neural networks potential (DP),^20,21^ which has been shown to yield an a-TiO_2_ bulk structure with equilibrium density and structural properties in good agreement with those experimentally obtained from the ALD deposited TiO_2_.^22^ We used cells with 3000 atoms and cooled down the system from 2250 to 300 K, with quenching rates of −1 K/ps. We then extracted 162 atoms from the 3000 atoms supercell and prepared a periodically repeated structure that was heated to 1000 K and cooled down again to 300 K to optimize its geometry (see the Supporting Information for details). We obtained an equilibrium density of ∼3.9 g/cm^3^, consistent with the values reported experimentally at room temperature (3.6–4.1 g/cm^3^).^23–25^ We compared radial distribution functions (RDFs) obtained with cells of 3000 and 162 atoms (see Figure S1) and found good agreement, indicating that the structure of a-TiO_2_ can be accurately represented even in small supercells.^18–20^ With both supercells, we found that nearly 40% of Ti atoms are undercoordinated (see Table S1). These results are consistent with those of a previous study reporting coordination numbers derived from X-ray absorption near edge spectroscopic data of ALD-prepared a-TiO_2_.^22^ The surface structure was obtained from the bulk a-TiO_2_ sample with a close-to-stoichiometric Ti:O ratio (see the Supporting Information for details).

#### Modeling BiVO_4_(010)

To prepare BiVO_4_(010) to be interfaced with a-TiO_2_, we approximated the monoclinic scheelite structure (*ms*-BiVO_4_) with a tetragonal scheelite (*ts*-BiVO_4_) geometry, as in our previous studies.^17,26,27^ Specifically, we used the *ms*-BiVO_4_ structure optimized with the SCAN functional (a = 5.122 Å, b = 5.128 Å, c = 11.568 Å, γ=90.0°, in a I2/b cell), whose geometry is close to that of the *ts*-BiVO_4_ structure. We used a slab with 6 BiVO_4_ layers terminated by a (010) 2 × 2 surface (*C*2/*c* representation); in a previous study we verified that such a slab is sufficiently thick to yield accurate results for the bandgap and band positions of BiVO_4_.^27^

#### Modeling BiVO_4_/a-TiO_2_ Interfaces

The a-TiO_2_ was interfaced with a stoichiometric BiVO_4_(010) surface and the interface geometry was investigated by carrying out FPMD simulations using density functional theory (DFT),^28^ with the strongly constrained and appropriately normed functional (SCAN).^29^ We used the Qbox code^30,31^ (versions 1.76.1, 1.76.4, 1.78.2) with Optimized Norm-Conserving Vanderbilt (ONCV)^32,33^ pseudopotentials and a 90 Ry energy cutoff. Constant temperature (T), NVT, and constant T and pressure (P), NPT, simulations were carried out by controlling the temperature of the system with the Bussi-Donadio-Parrinello thermostat.^34^ The time step was set at 20 au (i.e., ∼0.5 fs).

We prepared several BiVO_4_/a-TiO_2_ models to mimic the two BiVO_4_/TiO_2_ samples obtained experimentally. Specifically, we investigated a dry interface (with no water molecules present between the two solid oxides) and so-called wet interfaces, where one or two monolayers of water are present between the two solid oxides. For each model, the interface structure was heated at 400 K in NVT simulation for 4 ps, followed by an NPT simulation where only the cell dimension perpendicular to the interface (z) was optimized for 2 ps. The slab structure was then quenched to 300 K in NPT simulations and NVT simulations were conducted at 300 K for ∼5 ps. Geometries collected in the final ∼3 ps were used for the calculation of the local density of states (LDOS). For all LDOS plots reported in this study, the zero of energy in the absence of smearing is set at the average value of the VBM maxima of the a-TiO_2_ slab.

In the results and discussion section, we first present results for the electronic structure of the dry and wet interfaces at the SCAN level of theory, and we discuss trends in the electronic properties rather than absolute values of the energy gaps, which are known to be underestimated at this level of theory. We also performed calculations with a newly developed hybrid functional which accurately describes the structure of interfaces^35,36^ and found results for band-offsets in qualitative agreement with those of the SCAN functional, indicating the robustness of our results.

## Results and Discussion

### Experimental Investigation

#### Sample Preparation

We prepared two BiVO_4_/TiO_2_ samples that contain identical BiVO_4_ and TiO_2_ layers but have different interfacial structures. The BiVO_4_ layer was prepared as an epitaxial BiVO_4_(010) film using a previously reported method.^17,37^ (The *hkl* indices used in this study are based on the unit cell choice of a C-centered monoclinic cell, *C*2/*c*.^38^) Briefly, BiVO_4_ was grown on an yttrium-stabilized zirconia substrate covered with an epitaxially grown indium tin oxide (ITO) layer via pulsed layer deposition method. On the BiVO_4_(010) film, a ∼4 nm thick amorphous TiO_2_ layer was grown via atomic layer deposition (ALD), which is the most commonly used deposition method to coat a TiO_2_ protection layer on photoelectrodes. In ALD, TiO_2_ grows by alternately depositing a Ti layer using titanium tetraisopropoxide (TTIP) as the Ti source and an O layer using H_2_O as the O source. The total thickness of the TiO_2_ can be finely controlled by the number of cycles of Ti deposition and O deposition. When we deposited the TiO_2_ layer, we changed the sequence of Ti deposition and O deposition while keeping the total number of cycles the same. In one sample, referred to as BiVO_4_/TiO_2_(TTIP), we deposited the TiO_2_ layer on BiVO_4_ by introducing the Ti source first. In the other sample, referred to as BiVO_4_/TiO_2_(H_2_O), we deposited the TiO_2_ layer on BiVO_4_ by introducing the O source first. This ensured that while these two samples have identical BiVO_4_ and TiO_2_ layers, their interfacial structures are not identical. The TEM images of BiVO_4_(010)/TiO_2_(TTIP) and BiVO_4_(010)/TiO_2_(H_2_O) are shown in Figure 1a,b, where the same ∼4 nm thick TiO_2_ layer covering the BiVO_4_ surface is evident.

**Figure 1:**
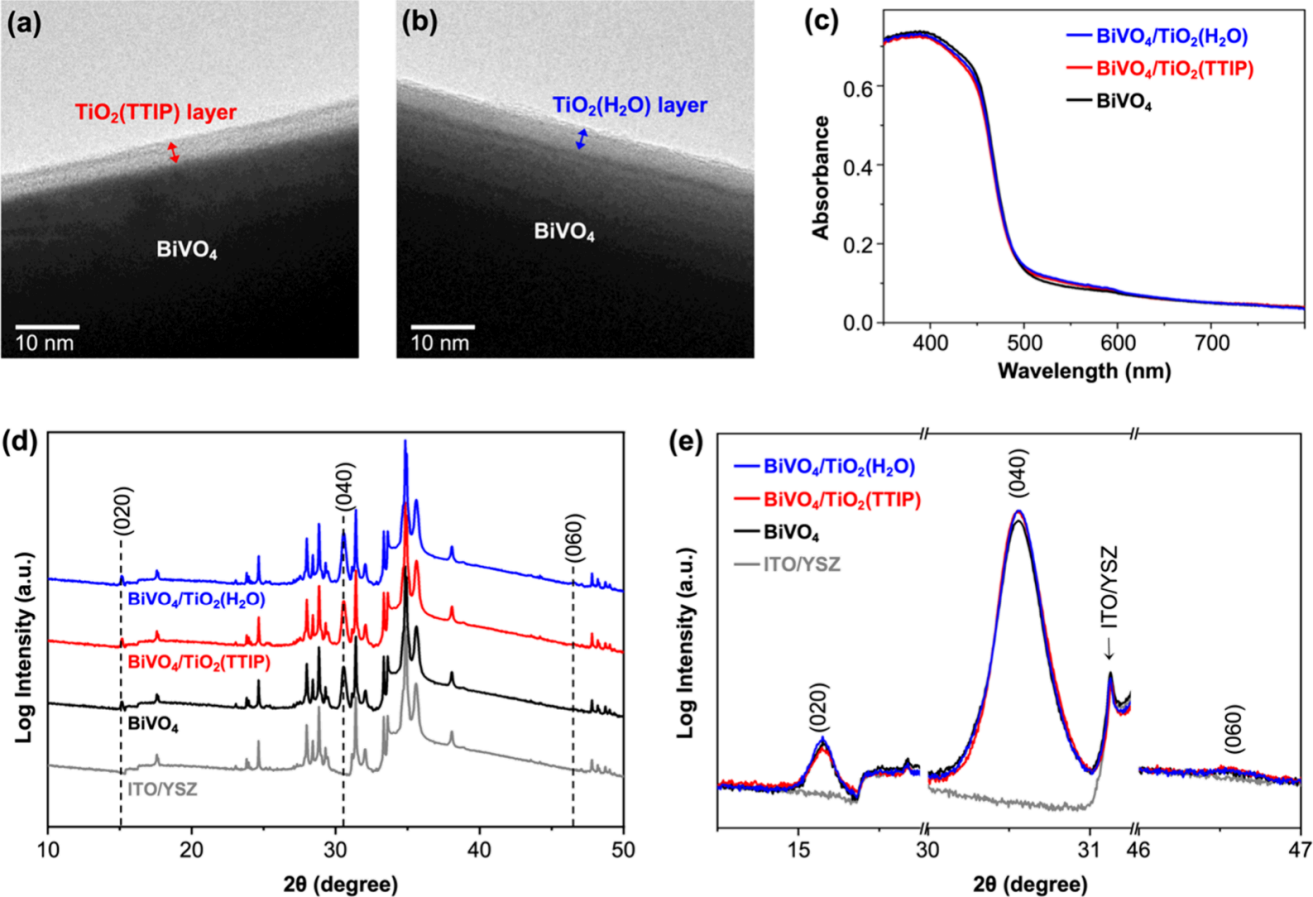
TEM images of (a) BiVO_4_/TiO_2_(TTIP) and (b) BiVO_4_/TiO_2_(H_2_O); (c) UV–vis spectra of BiVO_4_ (black), BiVO_4_/TiO_2_(TTIP) (red), and BiVO_4_/TiO_2_(H_2_O) (blue); (d) XRD patterns and (e) magnification of (0*k*0) diffraction peaks of ITO/YSZ (gray), BiVO_4_ (black), BiVO_4_/TiO_2_(TTIP) (red), and BiVO_4_/TiO_2_(H_2_O) (blue). The assigned (0*k*0) peaks are taken from JCPDS 14-0688.

UV–vis spectra of pristine BiVO_4_, BiVO_4_(010)/TiO_2_(TTIP), and BiVO_4_(010)/TiO_2_(H_2_O) are shown in Figure 1c. Their absorbances are identical, meaning the presence of a thin, wide-bandgap TiO_2_ film does not affect the absorption of BiVO_4_. The XRD pattern of the pristine BiVO_4_(010) film shows only (0*k*0) peaks, demonstrating that the BiVO_4_ used in this study is indeed an epitaxial film grown perpendicular to the [010] direction (Figure 1d,e). The XRD patterns of the two BiVO_4_(010)/TiO_2_ films show the identical XRD patterns to that of the pristine BiVO_4_(010) film, with no additional peaks from TiO_2_, which was expected as ALD-deposited TiO_2_ is known to be amorphous.^7–10,15,25^

We also analyzed the oxidation states of Ti in the TiO_2_ film in two BiVO_4_/TiO_2_ samples with Ti 2p XPS spectra (Figure 2).^10,15^ The peak fitting analysis shows that both samples contain the same amount of Ti^3+^ (∼5%). These characterization results confirm that the TiO_2_ layer in the two samples are identical in terms of thickness, crystallinity, and defect levels. Thus, if these two BiVO_4_/TiO_2_ samples show any difference in photoelectrochemical properties, it can be unambiguously attributed to the difference in the interfacial structure at the BiVO_4_/TiO_2_ junction.

**Figure 2:**
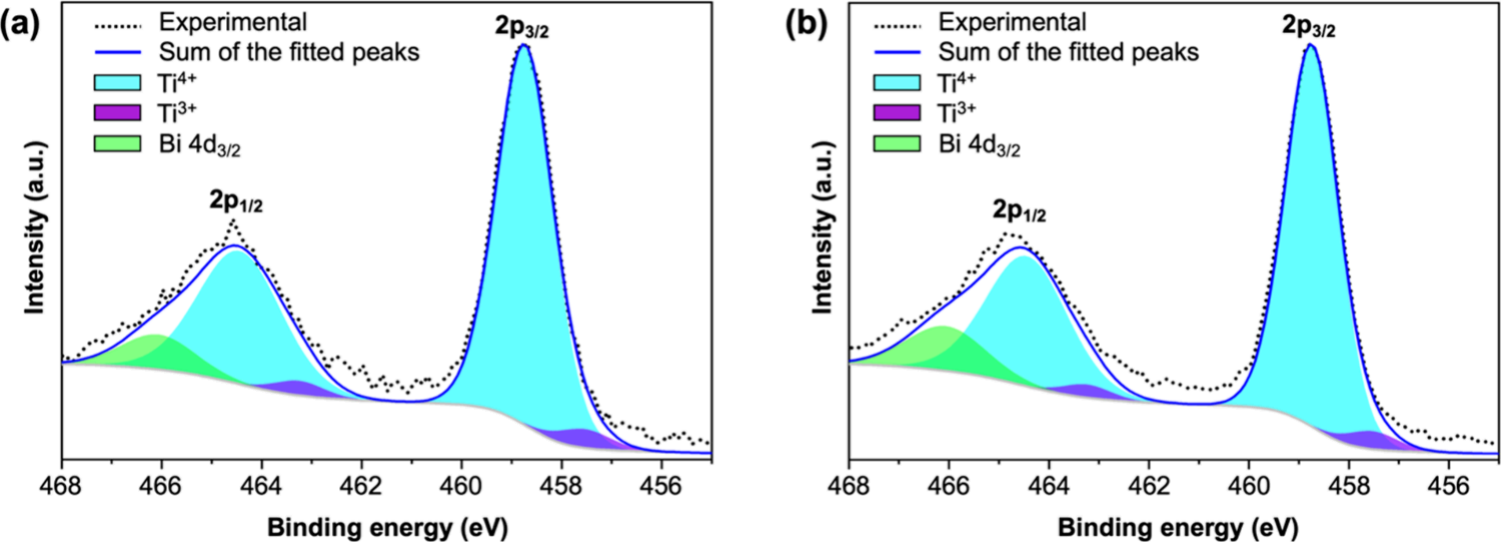
Ti 2p XPS spectra of (a) BiVO_4_/TiO_2_(TTIP) and (b) BiVO_4_/TiO_2_(H_2_O). The Ti^4+^ to Ti^3+^ ratios of both samples are 20:1, equivalent to ∼5% of the Ti present as Ti^3+^.

#### Photoelectrochemical Properties

The hole transfer from BiVO_4_ to TiO_2_ at the BiVO_4_/TiO_2_ interface of the two BiVO_4_/TiO_2_ samples was examined by measuring photocurrents for sulfite oxidation. Unlike water oxidation, which is kinetically slow and therefore loses a considerable amount of surface-reaching holes to surface recombination in the absence of an oxygen evolution catalyst, sulfite oxidation has fast kinetics that consumes all surface-reaching holes and suppresses surface recombination.^39,40^ Thus, the photocurrent measured for sulfite oxidation can be directly correlated to the number of holes reaching the photoanode surface, which is impacted by the degree of electron–hole recombination at the BiVO_4_/TiO_2_ interface in these two samples.

The *J–V* plots of pristine BiVO_4_, BiVO_4_/TiO_2_(TTIP), and BiVO_4_/TiO_2_(H_2_O) are shown in Figure 3. For each sample type, *J–V* plots were obtained using three different samples and the averaged *J–V* plots are shown. The individual *J–V* plots can be found in Figure S2. The pristine BiVO_4_ shows a higher photocurrent density than those of BiVO_4_/TiO_2_(TTIP) and BiVO_4_/TiO_2_(H_2_O), meaning considerable recombination occurs at the BiVO_4_/TiO_2_ interface if ALD-deposited TiO_2_ is interfaced with PLD-deposited epitaxial BiVO_4_(010) without further treatments to improve the quality of the interface. We note that the disorders and defects existing at the BiVO_4_/TiO_2_ interface may be removed by additional annealing procedures after TiO_2_ is added to BiVO_4_ by ALD. However, the goal of this study is not to make the best BiVO_4_/TiO_2_ interface to minimize the interfacial recombination. Instead, it is to investigate whether a subtle change of atomic structures at the interface caused by introducing the Ti source or O source first for TiO_2_ deposition can impact the interfacial band alignments. For this goal, using the as-prepared interfaces of BiVO_4_/TiO_2_(TTIP) and BiVO_4_/TiO_2_(H_2_O) without further modifications makes it easy to model the interfaces present in these samples and relate the property difference to the interfacial structure difference. Thus, we focus on comparing the performances of as-prepared BiVO_4_/TiO_2_(TTIP) and BiVO_4_/TiO_2_(H_2_O).

**Figure 3:**
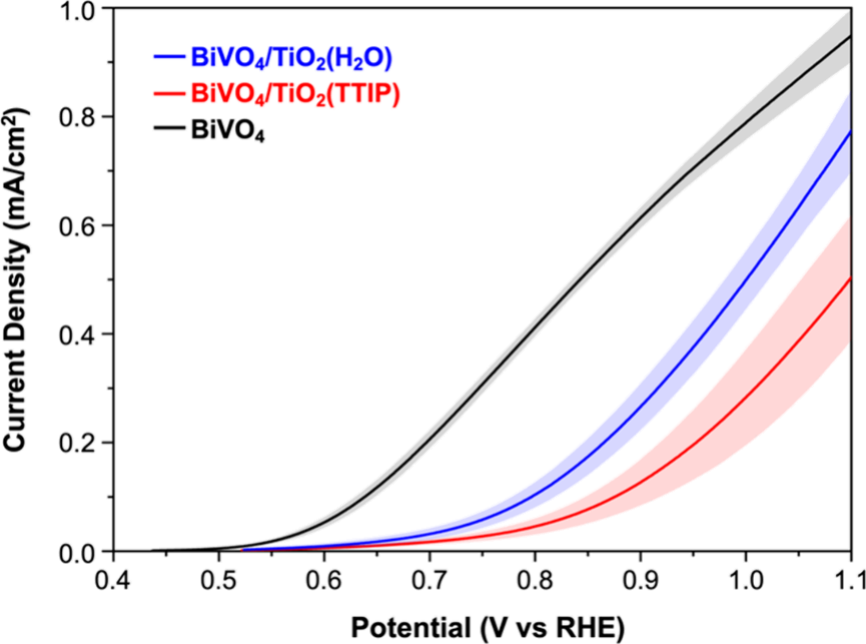
*J–V* plots for the sulfite oxidation of BiVO_4_ (black), BiVO_4_/TiO_2_(TTIP) (red), and BiVO_4_/TiO_2_(H_2_O) (blue) photoanodes measured in 0.5 M borate buffer (pH 9.3) containing 0.4 M sodium sulfite under AM1.5G illumination (back-side illumination). Shaded areas represent the standard deviation from the measurements of three different samples for each sample type.

In Figure 3, it is clearly shown that the photocurrent densities of BiVO_4_/TiO_2_(TTIP) and BiVO_4_/TiO_2_(H_2_O) show a meaningful difference. For example, the photocurrent density of BiVO_4_/ TiO_2_(H_2_O) at 0.9 V vs RHE is 0.27 ± 0.04 mA/cm^2^, which is two times higher than that of BiVO_4_/TiO_2_(TTIP) (0.13 ± 0.04 mA/cm^2^). Considering that the samples possess identical BiVO_4_ and TiO_2_ structures and differ only in the interfacial atomic structure, the observed performance difference is remarkable, and it clearly reveals the impact of the interfacial atomic structure on the overall photoelectrode performance. The meaningful performance difference was consistently observed with multiple samples (Figure S2).

In order to understand a microscopic-level origin of how using the Ti precursor or O precursor first to form a TiO_2_ layer on BiVO_4_ can affect interfacial band alignment and hole transfer at the BiVO_4_/TiO_2_ junction, we performed computational investigations using atomistic slab models of BiVO_4_(010) and amorphous TiO_2_ (a-TiO_2_) and interfacing them with plausible interfacial model structures.

### Computational Investigation

The surfaces of BiVO_4_(010) and a-TiO_2_ used to mimic the interfaces present in the BiVO_4_/TiO_2_(TTIP) and BiVO_4_/TiO_2_(H_2_O) samples are summarized in Figure 4. The interface present in the BiVO_4_/TiO_2_(TTIP) sample is relatively straightforward to model, by interfacing the dry surfaces of BiVO_4_(010) and a-TiO_2_ (Figure 4a). However, various considerations are needed to model the interface present in the BiVO_4_/TiO_2_(H_2_O) sample, due to multiple possibilities for H_2_O to be present at the BiVO_4_/TiO_2_ interface. We modeled several cases to mimic the variation in the amount and structure of water molecules at the BiVO_4_/a-TiO_2_ interface (Figure 4b), which are critical to comprehensively understand how the atomic structure at the interface affects the interfacial band offsets. The atomic and electronic structures of each model are discussed in detail below.

**Figure 4:**
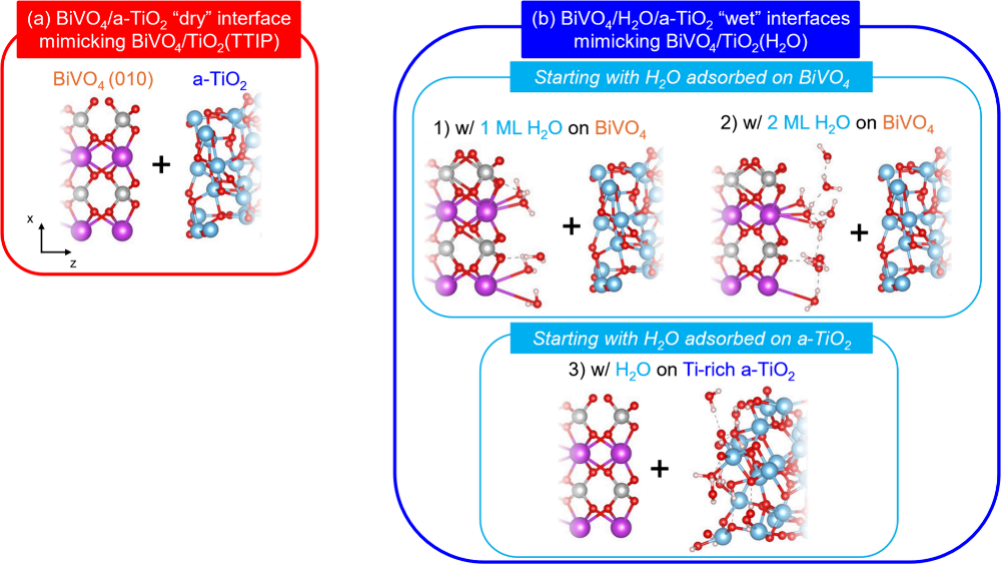
Structural models of BiVO_4_(010)/a-TiO_2_ interfaces investigated in this study, showing only the layers in proximity of the interface. Bi, V, Ti, O, and H atoms are represented by purple, gray, blue, red, and white spheres, respectively. (a) BiVO_4_/a-TiO_2_ “dry” interface with no water molecules between the two solid oxides mimicking the experimental sample of BiVO_4_/TiO_2_(TTIP). (b) BiVO_4_/H_2_O/a-TiO_2_ “wet” interfaces with water molecules between the two solid oxides mimicking the experimental sample of BiVO_4_/TiO_2_(H_2_O): (b1) starting with one monolayer (1 ML) of H_2_O initially adsorbed on the BiVO_4_ surface, (b2) starting with 2 ML of H_2_O initially adsorbed on the BiVO_4_ surface, and (b3) starting with H_2_O initially adsorbed on the Ti-rich a-TiO_2_ surface.

#### BiVO_4_/a-TiO_2_ Interface ("Dry" Interface)

We first describe the model used to mimic the interface present in the BiVO_4_/TiO_2_(TTIP) sample where the a-TiO_2_ layer was formed by introducing the Ti source first in the ALD procedure. When TTIP is introduced first, it will be adsorbed on the BiVO_4_ surface; the H_2_O introduced next will undergo hydrolysis and condensation reactions with the TTIP and form a Ti–O layer. By the subsequent introduction of the Ti and O sources, TiO_2_ layers will continuously grow. In this growth process, water molecules are highly unlikely to remain at the BiVO_4_/a-TiO_2_ interface and thus a ″dry″ BiVO_4_/a-TiO_2_ interface is expected.

Our computational model mimicking the dry interface was built by interfacing a stoichiometric BiVO_4_(010) surface and an a-TiO_2_ surface (Figure 4a) and performing FPMD simulations. The resulting energetically favored interfacial structures are shown in Figure 5a. Note that in our model the thickness of the a-TiO_2_ slab is ∼2 nm, which is smaller than but close to that of the experimentally prepared samples (∼4 nm) to offer qualitatively meaningful insights into the understanding of experimental samples. (Carrying out first-principles MD simulations with a 4 nm thick a-TiO_2_ slab would be computationally prohibitive.) At the BiVO_4_/a-TiO_2_ interface, each surface Bi atom of BiVO_4_ forms a chemical bond with one O atom belonging to a-TiO_2_, and each surface Ti atom of a-TiO_2_ forms a chemical bond with 1–2 O atoms belonging to the BiVO_4_ surface. Hence, the coordination numbers of surface Bi and Ti atoms are 7 and 5–6, respectively. These coordination numbers are close to those of bulk Bi and Ti atoms (8 and 5–6), indicating that there are no atoms with anomalously small coordination numbers in our interface model.

**Figure 5:**
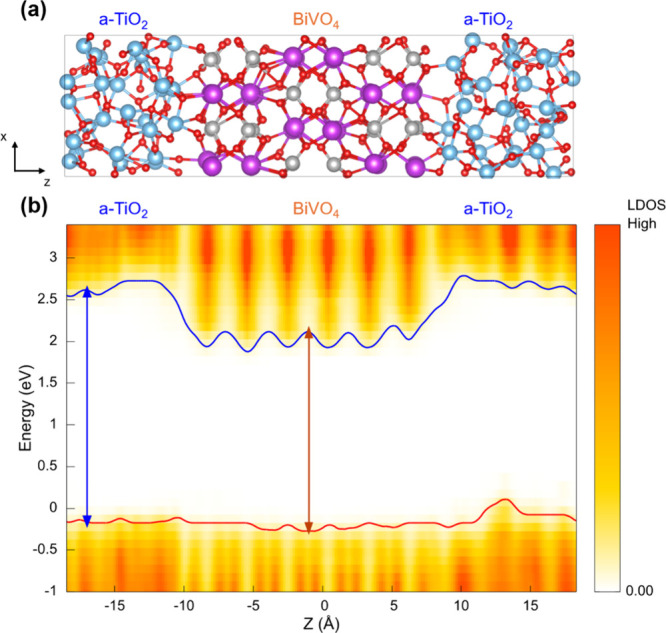
(a) Ball and stick structure of the BiVO_4_/a-TiO_2_ dry interface (same color coding of spheres as Figure 4a). (b) Local density of states (LDOS) along the direction perpendicular to the interface (*z* direction). The solid blue and red lines represent the position in energy of the CBM and VBM in the slab, respectively. The blue and red arrows indicate the average energy gap in the a-TiO_2_ and BiVO_4_ regions of the slab, respectively.

A computed LDOS, averaged from 30 frames of our simulation, is shown in Figure 5b. Our slab model is periodically repeated and therefore there are two BiVO_4_/a-TiO_2_ interfaces in the supercell. In addition, although the Ti:O ratio is similar on the two surfaces of a-TiO_2_, the structure and hence electronic structure of the two surfaces are not identical. The bandgaps of BiVO_4_ and TiO_2_ (i.e., the energy difference between the averaged CBM and averaged VBM for each material) are indicated by the red and blue arrows, respectively, in Figure 5b. We find that the CBM of the BiVO_4_ surface is located at a lower energy than that of a-TiO_2_ by ∼0.5 eV, and that the VBM of the two solid oxides are approximately at the same energy, indicating that the transfer of holes from BiVO_4_ to a-TiO_2_ is possible but not facile. We point out that the comparable VBM levels at the BiVO_4_/a-TiO_2_ interface obtained from our computational investigation are different from those predicted from the bulk band positions of BiVO_4_ and TiO_2_ where the VBM of TiO_2_ is lower than that of BiVO_4_ by ∼0.6 eV.^4,41^ This deviation indicates that the interfacial band alignment may not be accurately predicted by simply aligning the band positions of bulk materials.

To understand the sensitivity of the electronic structure to the atomic structure of a-TiO_2_, we prepared another a-TiO_2_ layer using the same procedure. Specifically, we extracted another 162-atom configuration from the initial 3000 atom bulk a-TiO_2_ generated by deep neural network potential molecular dynamics (DPMD);^20,21^ we then optimized the extracted structure using the method described in the SI and used the resulting a-TiO_2_ layer to form a BiVO_4_/a-TiO_2_ interface (Figure S3) different from that used originally. Due to the formation of chemical bonds at the interface, the coordination of Bi and Ti at the newly generated BiVO_4_/a-TiO_2_ interface is almost identical to that of the original interface shown in Figure 5. Also, the interfacial electronic structures — particularly the valence band offsets between BiVO_4_ and a-TiO_2_ at these two BiVO_4_/a-TiO_2_ interfaces — are almost identical (Figure 5b and Figure S3b).

We note that the BiVO_4_ and a-TiO_2_ slabs used here do not contain n-type defects and therefore the band alignments in the experimental samples may *quantitatively* deviate from our calculated results. However, the *qualitative* trend discussed in this study using interfacial atomic structures is expected to be accurate. Our future investigations will include the effects of n-type defects in both BiVO_4_ and TiO_2_ on the interfacial energetics.

#### BiVO_4_/H_2_O/a-TiO_2_ Interfaces ("Wet" Interfaces)

We now turn to describe the models built to mimic the BiVO_4_/TiO_2_(H_2_O) sample. When BiVO_4_/TiO_2_(H_2_O) is experimentally produced by introducing H_2_O as the O source first, the water molecules first adsorb on the BiVO_4_ surface. When the Ti precursor is introduced next, some of the surface adsorbed H_2_O molecules will react with TTIP to form an O–Ti bond via hydrolysis and condensation. However, the number of TTIP molecules adsorbed at the surface will be limited, due to the bulkiness of TTIP; hence not all H_2_O molecules adsorbed at the surface will be consumed by reacting with TTIP and some H_2_O molecules will remain intact. Upon further introduction of H_2_O and TTIP, TiO_2_ layers will continue growing on top of the first layer of TTIP, and the ″wet″ BiVO_4_/TiO_2_ interface can be preserved.

Previous studies have shown that the BiVO_4_(010) and the anatase TiO_2_ (001) surfaces in contact with liquid water exhibit an upward shift of their VBM toward the vacuum level by about 0.5 eV^27^ and 1.1 eV,^42^ respectively (both shifts were computed with hybrid functionals). A similar large shift of the VBM was also reported for the anatase surface when just one monolayer of molecularly adsorbed water is present on the surface.^42^ Such upward band edge shifts are due to a charge transfer from the O atom of molecularly adsorbed water to the surface cation (Bi in BiVO_4_ and Ti in TiO_2_), changing the surface polarization (Scheme S1a).^42,43^ On the other hand, when water adsorbs dissociatively, the charge transfer between the dissociated water species and the solid surface results in an opposite net surface polarization (Scheme S1b), causing the downward band edge shifts.

These results and considerations suggest that the interfacial band alignment may change depending on the following factors: (i) whether H_2_O is molecularly or dissociatively adsorbed; (ii) how many H_2_O molecules are adsorbed, and (iii) to which side of the interface (BiVO_4_ vs a-TiO_2_) H_2_O is more favorably adsorbed. Thus, to enable a comprehensive understanding of the effect of H_2_O at the interface, we built and examined three different BiVO_4_/H_2_O/a-TiO_2_ interface models. We start here by considering models in which water molecules are initially adsorbed on BiVO_4_.

##### Starting with H_2_O Adsorbed on BiVO_4_

A recent computational study of the BiVO_4_/water interface suggests that on the stoichiometric BiVO_4_(010) surface,^27^ H_2_O is preferably adsorbed in a molecular form. Thus, for models in which water molecules are initially adsorbed on BiVO_4_, we considered only molecularly adsorbed H_2_O. We constructed models with different amounts of H_2_O: one and two monolayers (ML). A one monolayer (1 ML) model contains five water molecules in our supercell; these 5 molecules are directly adsorbed on BiVO_4_ either on the surface Bi through the O of H_2_O or on the surface O through hydrogen bonded H of H_2_O (we refer to this model as BiVO_4_-1ML H_2_O/a-TiO_2_) (Figure 4b1). A two monolayer (2 ML) model includes five additional water molecules which form hydrogen bonds with the initial 1 ML water molecules directly adsorbed on the BiVO_4_ surface (we refer to this model as BiVO_4_-2ML H_2_O/a-TiO_2_) (Figure 4b2). We considered two models because the exact amount of H_2_O present in the experimental sample is unknown, and the amount of water may also vary depending on the deposition conditions. We then interfaced the two models of the H_2_O-adsorbed BiVO_4_ surfaces with a-TiO_2_ and carried out FPMD simulations.

The simulated interfacial structures of BiVO_4_-1ML H_2_O/a-TiO_2_ are presented in Figure 6a. While at the beginning of the simulation all H_2_O molecules are nondissociatively adsorbed on BiVO_4_, during the simulation we observed that some H_2_O molecules moved to the a-TiO_2_ surface, with both molecularly and dissociatively adsorbed H_2_O molecules eventually present on the a-TiO_2_ surface. The variations in the number of water molecules adsorbed molecularly and dissociatively on the BiVO_4_ and a-TiO_2_ surfaces during the simulation are shown in Figure S4.

**Figure 6:**
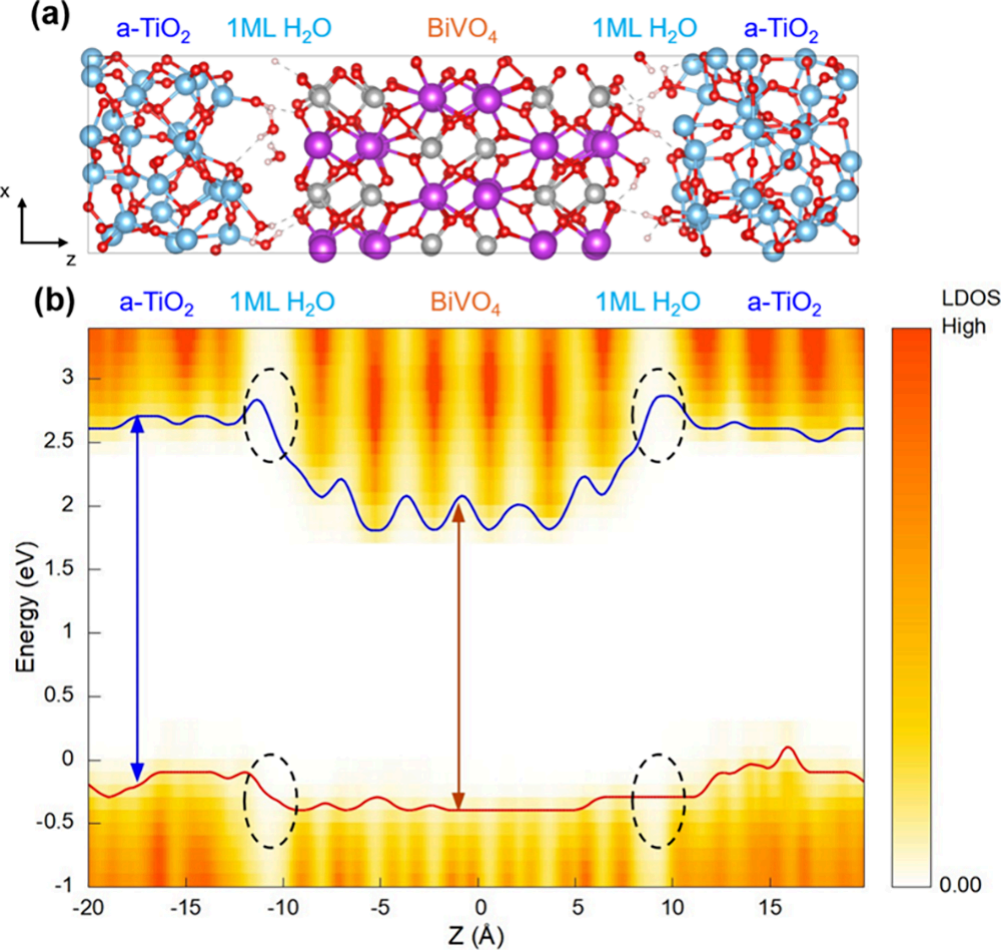
(a) Ball and stick representation of the BiVO_4_-1ML H_2_O/a-TiO_2_ interface (same color coding of spheres as Figure 4a). (b) LDOS along the direction perpendicular to the interface (*z* direction). The solid blue and red lines represent the position in energy of the CBM and VBM in the slab, respectively. The blue and red arrows indicate the energy gap in the a-TiO_2_ and BiVO_4_ regions of the slab, respectively. The black dashed circles indicate the BiVO_4_/a-TiO_2_ interfacial regions where the presence of water molecules led to a depletion of electronic states.

The resulting LDOS of BiVO_4_-1ML H_2_O/a-TiO_2_ (Figure 6b) differs from that of the dry interface in two major features. First, the VBM of a-TiO_2_ is higher in energy than that of BiVO_4_ by 0.2 eV. Both the VBMs of BiVO_4_ and TiO_2_ shift upward in the presence of molecularly adsorbed H_2_O; however, the degree of the shift is larger (by ∼0.6 eV^27,42^ ) for the VBM of TiO_2_. The upward shift of the VBM of TiO_2_ is compensated, in part, by a downward shift caused by the presence of dissociated water molecules at the a-TiO_2_ surface. The overall effect is that of positioning the VBM of a-TiO_2_ above that of BiVO_4_ by an amount (0.2 eV) smaller than 0.6 eV. However, the overall alignment remains favorable for hole transfer to the amorphous protective layer. Second, the presence of water molecules at the BiVO_4_/a-TiO_2_ interface leads to a depletion of electronic states in the BiVO_4_/a-TiO_2_ interfacial region (marked with black circles in Figure 6b) because the HOMO–LUMO gap of water is much larger than the bandgaps of a-TiO_2_ and BiVO_4_. Note that this depletion is more pronounced near the CBM than the VBM, because the energetic difference between the CBMs of BiVO_4_ and a-TiO_2_ and the LUMO of H_2_O is larger than that between the VBMs of BiVO_4_ and a-TiO_2_ and the HOMO of H_2_O. This depletion of states in proximity of the CBM at the BiVO_4_/a-TiO_2_ interface is favorable as it decreases the probability of electron tunneling from BiVO_4_ to a-TiO_2_, thereby decreasing electron–hole recombination of holes transferred to a-TiO_2_. Similar trends (although slightly less favorable) in terms of both the band alignment and the depletion of interfacial electronic states were observed when 2 ML of H_2_O are present (Figures S5 and S6), indicating that the presence of water at the interface can generally result in a more favorable interfacial band structures for hole transfer from BiVO_4_ to a-TiO_2_ than that of the ″dry″ interface.

Since our simulations with H_2_O initially adsorbed on BiVO_4_ showed some of the H_2_O molecules moving toward the a-TiO_2_ surface, we next considered a model in which water molecules are initially adsorbed on a-TiO_2_ to examine whether consistent results would be obtained.

##### Starting with H_2_O Adsorbed on a-TiO_2_

In our model of a-TiO_2_ surfaces containing adsorbed water molecules, we considered a Ti-rich surface, since the introduction of TTIP after H_2_O leads to the growth of an a-TiO_2_ layer with excess Ti atoms exposed at the BiVO_4_/H_2_O/a-TiO_2_ interface. The Ti-rich a-TiO_2_ surface was obtained by starting from the bulk amorphous structure and extracting a slab with a surface having a Ti:O ratio higher than 1:2 (i.e., a Ti:O ratio of ∼1:1). In this procedure, we did not intentionally remove any O from the surface. Thus, while this slab contains a Ti-rich surface, the stoichiometry of this slab is the same as that of the regular a-TiO_2_ slab with the Ti:O ratio of 1:2. We then interfaced the Ti-rich a-TiO_2_ surface described above with 64 water molecules representing a sample of bulk water, and we carried out FPMD simulations at room temperature (see the SI for additional information regarding the Ti-rich a-TiO_2_ surface). These simulations were performed to first determine whether water would adsorb nondissociatively or dissociatively on the Ti-rich a-TiO_2_ surface. The number of water molecules adsorbed on the surface during the simulation is shown in Figure S7. The results show that approximately eight H_2_O molecules are directly adsorbed on the surface, with five of them adsorbed nondissociatively and three of them adsorbed dissociatively.

As the number of H_2_O molecules directly adsorbed on the Ti-rich a-TiO_2_ surface is more than that on BiVO_4_ (i.e., five used in BiVO_4_-1ML H_2_O/a-TiO_2_), in order to make a fair comparison with BiVO_4_-2ML H_2_O/a-TiO_2_ having ten H_2_O molecules, we introduced two more H_2_O molecules to the Ti-rich a-TiO_2_ surface and then interfaced the resulting TiO_2_ surface with BiVO_4_ to perform FPMD simulations. The resulting interface model is referred to as BiVO_4_/H_2_O-a-(Ti)­TiO_2_.

The simulated interface and LDOS results of BiVO_4_/H_2_O-a-(Ti)­TiO_2_ are shown in Figure 7. The number of H_2_O molecules adsorbed on a-TiO_2_ and BiVO_4_ both molecularly and dissociatively during the simulation can be found in Figure S8.

**Figure 7:**
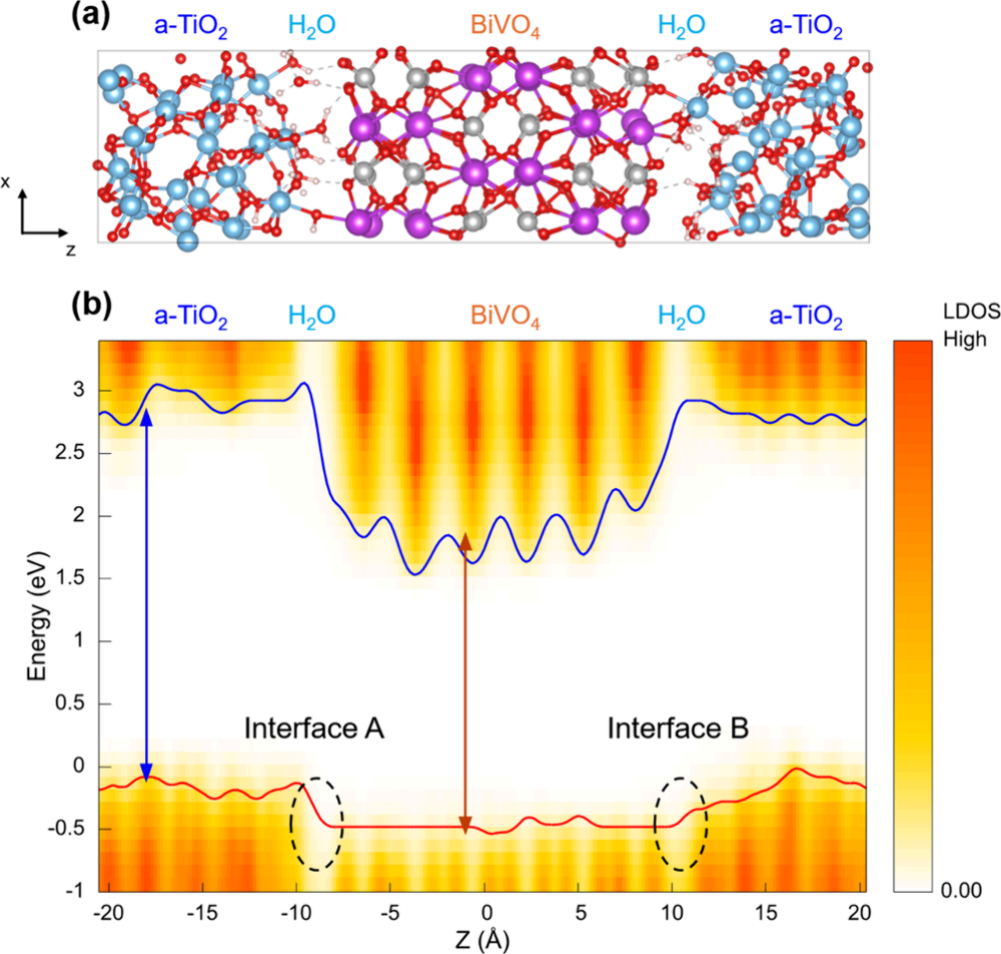
(a) Ball and stick representation of the BiVO_4_/H_2_O-a-(Ti)­TiO_2_ interface structures (same color coding of spheres as Figure 4a) using the Ti-rich a-TiO_2_ interface. (b) LDOS along the direction perpendicular to the interface (*z* direction). The solid blue and red lines represent the position in energy of the CBM and VBM in the slab, respectively. The blue and red arrows indicate the energy gap in the a-TiO_2_ and BiVO_4_ regions of the slab, respectively. The black dashed circles indicate the BiVO_4_/a-TiO_2_ interfacial regions where the presence of water molecules affects the density of the electronic states near the VBM.

The comparison between the interfacial water adsorption in the BiVO_4_/H_2_O-a-(Ti)­TiO_2_ (Figure 7) and BiVO_4_-2ML H_2_O/a-TiO_2_ models (Figure S5), both of which possess ten H_2_O molecules at the interface, shows two clear differences. Both the average numbers of H_2_O molecularly and dissociatively adsorbed on the TiO_2_ side are larger for BiVO_4_/H_2_O-a-(Ti)­TiO_2_ (Table S2). The difference in the number of dissociated water molecules mainly comes from the fact that BiVO_4_/H_2_O-a-(Ti)­TiO_2_ has a Ti-rich a-TiO_2_ surface while BiVO_4_-2 MLH_2_O/a-TiO_2_ has a-TiO_2_ surface with a Ti:O ratio close to the stoichiometric one.

The structural differences between the two models result in two critical differences in the electronic structure of the interface. First, the VBM of a-TiO_2_ is located 0.4 eV above that of BiVO_4_ in BiVO_4_/H_2_O-a-(Ti)­TiO_2_ (Figure 7b) while the VBM of a-TiO_2_ is located 0.2 eV above that of BiVO_4_ in BiVO_4_-2 MLH_2_O/a-TiO_2_ (Figure S5b). This difference originates from a larger number of H_2_O adsorbed molecularly on the a-TiO_2_ side in BiVO_4_/H_2_O-a-(Ti)­TiO_2_, thus increasing the degree of upward shift of the VBM of a-TiO_2_. (See also wave function analysis provided in Figure S9.) Second, the density of states of H_2_O at the BiVO_4_/a-TiO_2_ junction near the VBM of a-TiO_2_ is higher in BiVO_4_/H_2_O-a-(Ti)­TiO_2_ (i.e., compare black circled regions in Figure 7b and Figure S5b in the VBM). This is due to a higher number of H_2_O dissociatively adsorbed on the a-TiO_2_ side in BiVO_4_/H_2_O-a-(Ti)­TiO_2_. The higher density of states near the VBM of a-TiO_2_ can facilitate the transfer of holes from BiVO_4_ and can contribute to the decrease in interfacial recombination at the BiVO_4_/a-TiO_2_ junction. The effect of H_2_O dissociatively adsorbed on a-TiO_2_ in altering the density of states near the VBM of a-TiO_2_ can also be seen by comparing the two BiVO_4_/a-TiO_2_ interfaces (Interface A and Interface B) present in the slab modeling BiVO_4_/H_2_O-a-(Ti)­TiO_2_, shown in Figure 7. Among the two BiVO_4_/TiO_2_ interfaces, the LDOS shows a higher density of states from H_2_O near the VBM of TiO_2_ at Interface B because the number of dissociatively adsorbed H_2_O is higher at Interface B (Figure S8).

In sum, the higher numbers of both molecularly and dissociatively adsorbed water molecules to the TiO_2_ side in BiVO_4_/H_2_O-a-(Ti)­TiO_2_ offer more favorable interfacial energetics for hole transfer from BiVO_4_ to TiO_2_ than that in BiVO_4_-2ML H_2_O/a-TiO_2_.

Figure 8 summarizes the band alignments of interfacial models computationally investigated in this study at the SCAN level of theory. Our results show that the three “wet” interfaces mimicking the experimental BiVO_4_/TiO_2_(H_2_O) sample consistently show more favorable interface energetics for hole transfer from BiVO_4_ to TiO_2_ than the “dry” interface used to mimic the experimental BiVO_4_/TiO_2_(TTIP) sample. From these results, we conclude that the presence of water molecules at the BiVO_4_/a-TiO_2_ interface can generally promote hole transfer from BiVO_4_ to a-TiO_2_.

**Figure 8:**
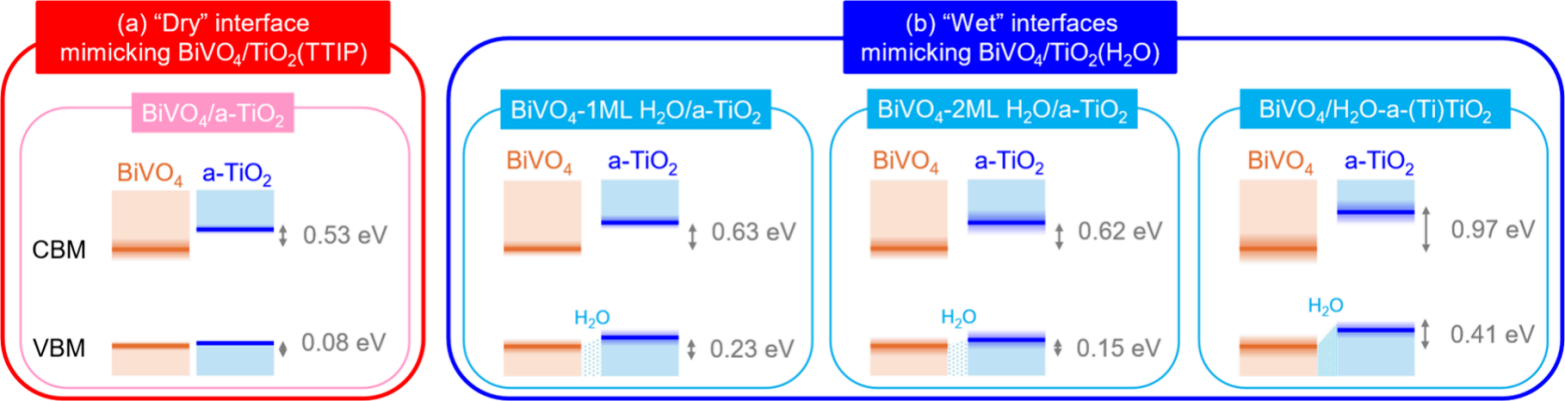
Band alignment of various BiVO_4_/a-TiO_2_ models after 300 K FPMD simulations, as obtained from SCAN calculations. Numbers in gray indicate band offset values. For the VBM and CBM, the shading around the band edges represents temperature fluctuations in the energy eigenvalue, as estimated from FPMD snapshots.

Finally, in order to validate our results obtained at the SCAN level of theory, we also carried out additional calculations with hybrid functionals.^35,36^ The electronic structures of the slabs were calculated for one snapshot structure obtained from the simulations of the BiVO_4_/a-TiO_2_ “dry” interface and the BiVO_4_/H_2_O-a-(Ti)­TiO_2_ “wet” interface, respectively. The results are shown in Figure 9. (For consistency with our SCAN results, the band gaps were not corrected in the figure for nuclear quantum effects, spin–orbit coupling, and exciton contributions; if such corrections are applied,^26^ the computed bandgap agrees well with the experimental values. Also note that the bandgap of a-TiO_2_ is found to be close to the fundamental band gap of crystalline anatase TiO_2_ if we take its zero-point renormalization into account.^44^). Importantly, our results with hybrid functionals show the same trends for band offsets as those computed with the SCAN functional: the VBM energy of a-TiO_2_ is much higher than that of BiVO_4_ in BiVO_4_/H_2_O-a-(Ti)­TiO_2_ (note that this trend holds even after the renormalization is applied on BiVO_4_). Therefore, the conclusions obtained with the SCAN functional are validated by the results of electronic structure calculations using the hybrid functional.

**Figure 9:**
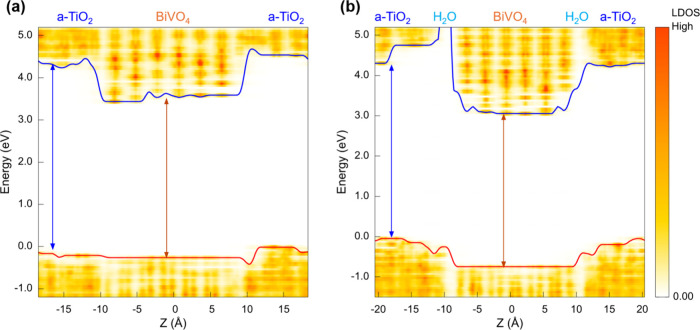
Local density of states (LDOS) of (a) BiVO_4_/a-TiO_2_ “dry” interface and (b) BiVO_4_/H_2_O-a-(Ti)­TiO_2_ “wet” interface obtained using a hybrid functional. The solid blue and red lines represent the position in energy of the CBM and VBM in the slab, respectively. The blue and red arrows indicate the energy gap in the a-TiO_2_ and BiVO_4_ regions of the slab, respectively.

### Experimental Verification of the Computational Results

Our computational result explained that the performance difference between BiVO_4_/TiO_2_(TTIP) and BiVO_4_/TiO_2_(H_2_O) originated from the presence of H_2_O in BiVO_4_/TiO_2_(H_2_O) affecting the interfacial band alignment and hole transfer. Thus, we attempted to probe the presence of H_2_O in BiVO_4_/TiO_2_(H_2_O) using O 1s XPS. The detection of H_2_O present at the BiVO_4_/TiO_2_ interface can be extremely challenging as this interface is buried under a ∼4 nm-thick TiO_2_ layer and O from the TiO_2_ layer can dominate the XPS spectra. Thus, we prepared BiVO_4_/TiO_2_(TTIP) and BiVO_4_/TiO_2_(H_2_O) samples with an extremely thin TiO_2_ layer by using 5 ALD cycles instead of the 123 cycles used to form a ∼4 nm thick TiO_2_ layer, so that we can probe the presence of H_2_O at the interface using O 1s XPS with less interference from O in the TiO_2_ layer. The O 1s XPS spectra of BiVO_4_/TiO_2_(TTIP) and BiVO_4_/TiO_2_(H_2_O) are shown in Figure 10.

**Figure 10:**
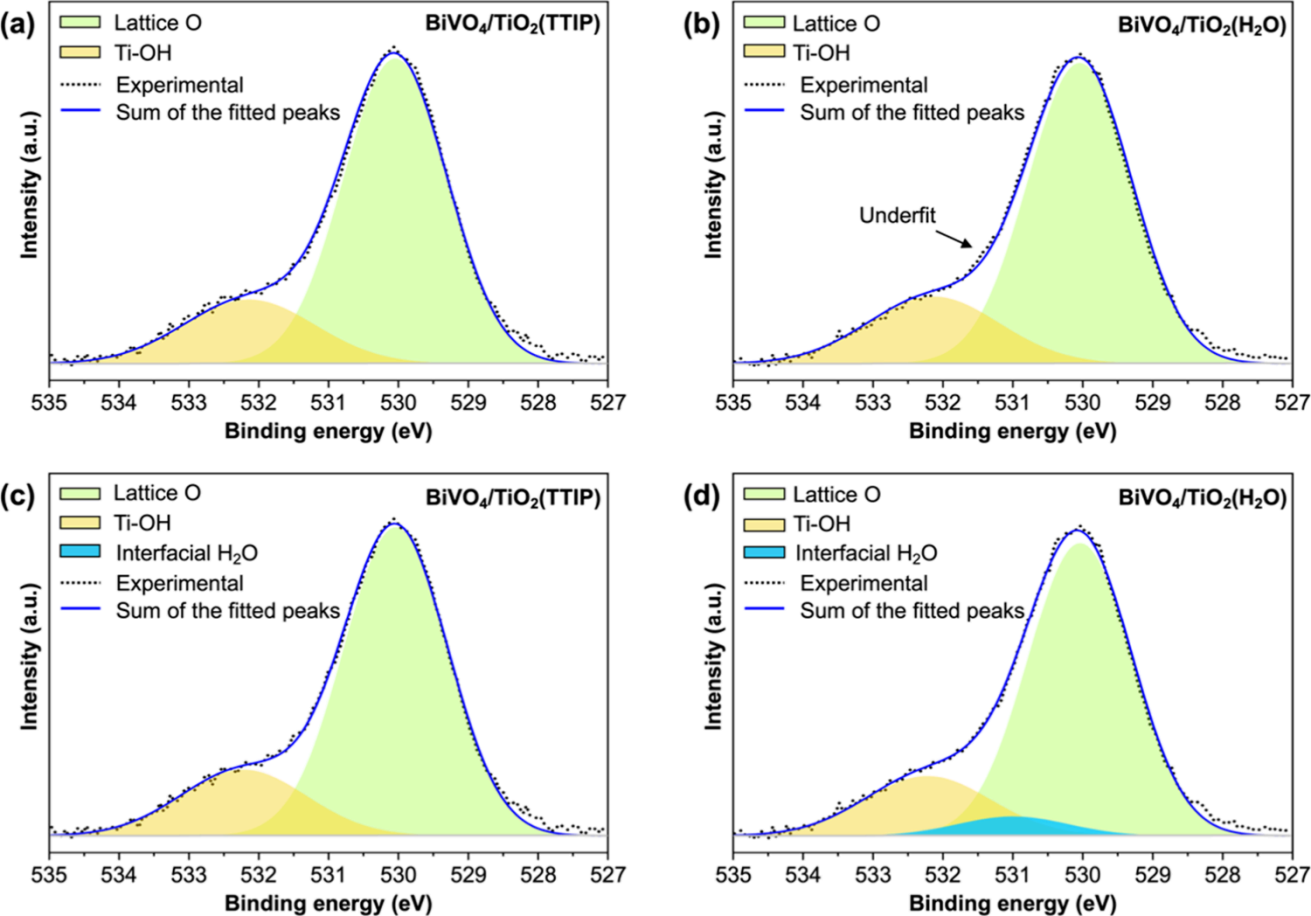
Deconvolution of O 1s XPS spectra of (a) BiVO_4_/TiO_2_(TTIP) and (b) BiVO_4_/TiO_2_(H_2_O) using two peaks (lattice O and surface Ti–OH). Deconvolution of O 1s XPS spectra of (c) BiVO_4_/TiO_2_(TTIP) and (d) BiVO_4_/TiO_2_(H_2_O) using three peaks (lattice O, surface Ti–OH, and interfacial H_2_O).

Initially, we deconvoluted the O 1s peak into two peaks: one centered at 530.0 eV, which is due to lattice O; and the other centered at 532.1 eV, which is due to surface Ti–OH (Figure 10a,b).^45^ However, we noticed an underfit region on the left side of the lattice O peak of BiVO_4_/TiO_2_(H_2_O), where the simulated intensity is less than the observed intensity, while the same region of the BiVO_4_/TiO_2_(TTIP) sample shows a good fit. Thus, we attempted to add one more peak between the two peaks of BiVO_4_/TiO_2_(TTIP) and BiVO_4_/TiO_2_(H_2_O) (Figure 10c,d). Indeed, the O 1s peak of BiVO_4_/TiO_2_(H_2_O) was better fitted with three peaks, with the third peak centered at 531.0 eV. For the case of BiVO_4_/TiO_2_(TTIP), the weight of the third peak went to zero, meaning the O 1s peak of this sample was optimally fitted with two peaks and there was no residue to accommodate an additional peak. The detailed fitting parameters and results are summarized in Tables S3 and S4.

The third peak centered at 531.0 eV present only in BiVO_4_/TiO_2_(H_2_O) has been reported to be caused by strongly chemisorbed H_2_O, where the O atom in H_2_O forms a dative bond to the surface metal cation and the H atom in H_2_O forms a hydrogen bond to surface O.^46^ The description of chemisorbed H_2_O matches well with H_2_O present at the BiVO_4_/TiO_2_ interface in BiVO_4_/TiO_2_(H_2_O) where the O of some H_2_O molecules forms a dative bond to surface Bi and the H of those H_2_O molecules forms a hydrogen bond to the surface O of BiVO_4_ or surface O of TiO_2_ (Figures 6a and 7a). Thus, this third peak present only in BiVO_4_/TiO_2_(H_2_O) strongly supports that our experimental BiVO_4_/TiO_2_(H_2_O) sample possesses interfacial H_2_O predicted by the computational results.

We also employed electrochemical impedance spectroscopy (EIS) to examine how the difference in interfacial hole transfer at the BiVO_4_/TiO_2_ interface in BiVO_4_/TiO_2_(TTIP) and BiVO_4_/TiO_2_(H_2_O) samples manifests in their Nyquist plots. The comparison of their Nyquist plots with that of BiVO_4_ reveals that only the semicircle appearing in the low frequency region is caused by the addition of the TiO_2_ layer (Figure 11). When we analyzed these low frequency semicircles to obtain the charge transfer resistance (*R*_CT_) and charging capacitance (*C*_IT_) caused by the addition of the TiO_2_ layer using an equivalent circuit model shown in the inset of Figure 11 (details in Figure S10), the *R*_CT_ value of BiVO_4_/TiO_2_(TTIP) was notably higher than that of BiVO_4_/TiO_2_(H_2_O) (Table S5). This was expected as BiVO_4_/TiO_2_(TTIP) showed a much lower photocurrent density (Figure 3). It is interesting to note that the low photocurrent generation of BiVO_4_/TiO_2_(TTIP) due to a higher recombination loss of holes at the BiVO_4_/TiO_2_ interface appears as a higher *R*_CT_ in EIS analysis. What is revealing is that the *C*_IT_ values of the two samples were comparable (Table S5). This indicates that the two samples possess comparable amounts of interfacial states that can serve as hole traps and therefore the higher recombination loss of holes at the interface in the BiVO_4_/TiO_2_(TTIP) sample is not due to a difference in the density of interfacial states. Thus, although our current computational studies did not consider the formation of interfacial trap states, our explanation of interfacial hole transfer and recombination based on the interfacial band alignments appears to be sufficient to understand the experimental results.

**Figure 11:**
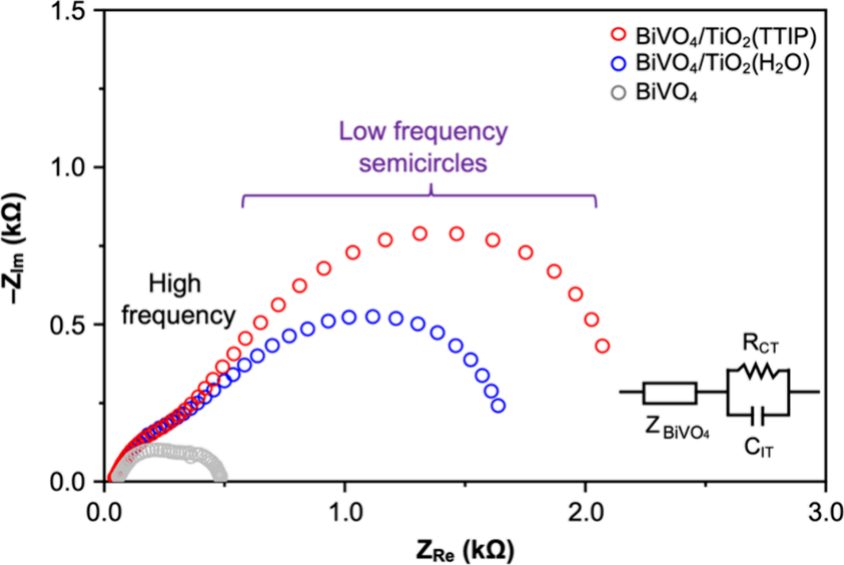
Nyquist plots of BiVO_4_, BiVO_4_/TiO_2_(TTIP), and BiVO_4_/TiO_2_(H_2_O) measured at 0.7 V vs RHE under the same conditions used to obtain the *J–V* plots shown in Figure 3. The inset shows an equivalent circuit used to obtain *R*_CT_ and *C*_IT_ values shown in Table S5. *Z*_BiVO4_ is the impedance originating from the common components of the BiVO_4_/TiO_2_(TTIP) and BiVO_4_/TiO_2_(H_2_O) samples (e.g., BiVO_4_ and the BiVO_4_/ITO back contact).

## Conclusions

In this study, we examined the impact of interfacial atomic structures and bonding at the BiVO_4_(010)/a-TiO_2_ interface on the interfacial electronic structures and hole transfer from BiVO_4_ to TiO_2_ in BiVO_4_/TiO_2_ photoanodes. BiVO_4_/TiO_2_(TTIP) and BiVO_4_/TiO_2_(H_2_O) samples were prepared by changing the sequence of introducing the Ti source (TTIP) and O source (H_2_O) during the ALD growth of TiO_2_ while keeping the other features of the BiVO_4_ layer and TiO_2_ layer identical. Although the difference between these two photoanodes was only the interfacial atomic structure, the BiVO_4_/TiO_2_(H_2_O) sample consistently generated higher anodic photocurrent than BiVO_4_/TiO_2_(TTIP), suggesting the formation of a more favorable band alignment for hole transfer from BiVO_4_ to TiO_2_. In order to elucidate the atomic origin of the observed performance difference in these two samples, plausible interfacial structures present in these two samples were computationally investigated. To mimic the interface present in BiVO_4_/TiO_2_(TTIP), a ″dry″ interface was simulated by interfacing BiVO_4_(010) and a-TiO_2_ using FPMD simulations. To mimic the interface present in BiVO_4_/TiO_2_(H_2_O), a ″wet″ interface was simulated where H_2_O molecules are presented at the BiVO_4_/a-TiO_2_ junction, recognizing that it is conceivable that not all H_2_O molecules first introduced on the BiVO_4_ surface are consumed by the monolayer of TTIP introduced subsequently due to the bulkiness of TTIP. Three different ″wet″ interfaces were considered where the number of H_2_O molecules, Ti:O ratio of the a-TiO_2_ surface interfacing with BiVO_4_, and the initial location of H_2_O before FPMD simulations were varied. Both the ″dry″ and ″wet″ interfaces show that the VBM alignment between BiVO_4_ and a-TiO_2_ is more favorable than that expected by the VBMs of bulk BiVO_4_ and TiO_2_ when a thin a-TiO_2_ layer is used. Furthermore, while there was a slight variation within the ″wet″ interfacial models, all the ″wet″ interfacial models resulted in more favorable interfacial electronic structures than that of the ″dry″ interface in terms of the VBM alignment and depletion of the electronic states near the CBM at the BiVO_4_/TiO_2_ interface, both of which can reduce interfacial electron–hole recombination. These results unambiguously show the effect of the interfacial atomic structures on the interfacial electronic structures and explain the experimentally observed differences of the two samples. More broadly, considering that there are multiple synthesis methods to deposit a TiO_2_ protection layer on semiconductor electrodes, where some are dry methods (e.g., sputter coating and pulsed laser deposition) and some are wet methods (e.g., electrodeposition and sol–gel method), our results provide new insights that the synthesis method of TiO_2_ can affect the interfacial electronic structures not only by changing the quality of the TiO_2_ layer (crystallinity, defect level) but also by changing the interfacial atomic structure and bonding at the semiconductor/TiO_2_ junction.

## Supplementary Material



## Notes

The Supporting Information is available free of charge on the ACS Publication Web site: Additional *J–V* plots, XPS and EIS fitting results, additional computational methods, a scheme showing surface dipoles resulting from molecularly and dissociatively adsorbed water molecules, the number of water molecules adsorbed on BiVO_4_ side or TiO_2_ side during the simulations for various BiVO_4_/TiO_2_ interface models, LDOS results of the BiVO_4_-2ML H_2_O/TiO_2_ interface, an iso-surface of wave functions near VBM of BiVO_4_/H_2_O-a-(Ti)­TiO_2_ interface structure, and control calculations involving a-(Ti)­TiO_2_ (PDF)
