# Supplementary material for: Impact of the Atomic Structure at the BiVO4/TiO2 Interface on the Electronic Properties and Performance of BiVO4/TiO2 Photoanodes
Source: J Am Chem Soc. 2025 Aug 14;147(34):30851–62. doi: 10.1021/jacs.5c07695 (PMC12503355; doi:10.1021/jacs.5c07695)
Supplement: Supplementary file 1 [file ja5c07695_si_001.pdf]

Supporting Information

Impact of the Atomic Structure at the $BiVO_4/TiO_2$ Interface on the Electronic Properties and Performance of $BiVO_4/TiO_2$ Photoanodes

Dae Han Wi,[1,2,#] Kana Ishisone,[3,#] Zhaoyi Xi,[4,5,#] Zifan Ye,[3] Daye Seo,[1] Jiawei Zhan,[3] Xiao Tong,[4] Mingzhao Liu,[4,*] Giulia Galli,[3,6,7*] and Kyoung-Shin Choi[1,*]

[1]*Department of Chemistry, University of Wisconsin-Madison, Madison, WI 53706, United States*
[2]*Department of Chemistry, Chungnam National University, Daejeon 34134, Republic of Korea*
[3]*Pritzker School of Molecular Engineering, University of Chicago, Chicago, IL 60637, USA*
[4]*Center for Functional Nanomaterials, Brookhaven National Laboratory, Upton, NY 11973, United States*
[5]*Department of Materials Science and Chemical Engineering, Stony Brook University, Stony Brook, NY 11794, United States*
[6]*Department of Chemistry, University of Chicago, Chicago, IL 60637, USA*
[7]*Argonne National Laboratory, Lemont, IL 60439, USA*

[#] These authors contributed equally.
* Correspondence and requests for materials should be addressed to M.L (email: mzliu@bnl.gov), G.G. (email: gagalli@uchicago.edu), and K.-S.C. (email: kschoi@chem.wisc.edu).

Additional computational methods

Structural models of amorphous TiO_2 (a-TiO_2) using a deep neural networks potential. Recently, deep neural networks potentials (DPs) were developed to model bulk a-TiO_2 and the system in contact with bulk water.[S1,S2] In this study, we generated the initial structure of a-TiO_2 using molecular dynamics simulations using the DPs (DPMD) of Refs. S1-S2. The simulations were performed with the Large-scale Atomic/Molecular Massively Parallel Simulator (LAMMPS) code interfaced with the DeePMD-kit.[S3,S4] We initially used a cell of 3000 atoms for TiO_2, melted the system by bringing it to a T of 2500 K for 1 ns, followed by a quenching cycle from 2250 K to 300 K carried out with NPT simulations at the rate of -1 K/ps. We further equilibrated the structure at 300 K for 1 ns. We then extracted a smaller sample of 162 atoms from the large 3000 atom cell, commensurate with the $BiVO_4$(010) 2x2 slab used in our study. Finally, the 162 atom structure was heated up to 1000 K to optimize its geometry, followed by a quenching to 300 K with NPT simulations where we only optimized the direction (z) perpendicular to the interface; the cell components in the x- and y- directions were kept constant. The radial distribution functions (RDFs) and Ti coordination numbers obtained in our calculations are given in **Figure S1** and **Table S1**, respectively, and show good agreement with experiments, indicating that the structure of a-TiO_2 can be accurately represented even in small supercells. With both supercells, we found that nearly 40% of Ti atoms are undercoordinated (**Table S1**). These results are consistent with those of a previous studies reporting coordination numbers derived from X-ray absorption near edge spectroscopical data of ALD-prepared a-TiO_2.[S5] The surface structure was obtained from the bulk a-TiO_2 structure with the Ti:O ratio on the surface close to the stoichiometric ratio. The preparation of a Ti-rich a-TiO_2 surface slab is described in the following section.

Interfaces between the Ti-rich a-TiO_2 and water. The Ti-rich a-TiO_2 surface was obtained by starting from the bulk amorphous structure prepared by the method described above and extracting a slab with a surface having a Ti:O ratio higher than 1:2 (i.e., a Ti:O ratio of ~1:1) from the periodically repeated bulk model. In this procedure, we did not intentionally remove any O from the surface. Thus, while this slab contains a Ti-rich surface, the stoichiometry of this slab is the same as that of a regular TiO_2 slab with a Ti:O ratio of 1:2. We then interfaced the surface of a-(Ti)TiO_2 with 64 water molecules and carried out first principles NPT simulations (by optimizing only the z-component of the slab) at 330 K. These calculations were followed by NVT simulations using DPMD for 2.5 ns at 300 K.[S2] The number of water molecules adsorbed on each surface during the simulation is shown in **Figure S6**.

The electrostatic potential of $BiVO_4$/H_2O-a-(Ti)TiO_2 is shown in **Figure S11**, which suggests that there is no significant total dipole moment across the entire simulation cell. We also compared the existence of localized states at the $BiVO_4$/a-TiO_2 and $BiVO_4$/H_2O-a-(Ti)TiO_2 interfaces due to defects such as Ti^{3+} (**Figure S12**). In both cases, localized levels are present at 0.2-0.3 eV above the VBM, and no significant difference in the density of localized levels is observed between the two samples. This result indicates that the difference in band alignment between $BiVO_4$/a-TiO_2 and $BiVO_4$/H_2O-a-(Ti)TiO_2 discussed in the main text is not due to the difference in the amount of defects (e.g., Ti^{3+}) or localized levels at the interface, but due to the presence of interfacial water in $BiVO_4$/H_2O-a-(Ti)TiO_2. We also additionally constructed a *dry* $BiVO_4$/a-(Ti)TiO_2 interface to confirm that the more favorable interfacial band alignment of $BiVO_4$/H_2O-a-(Ti)TiO_2 is from the presence of interfacial water interacting with the Ti-rich TiO_2 surface and not from the Ti-rich TiO_2 surface alone (**Figure S13**).

Table S1. Fraction (in %) of n-coordinated (c) Ti atoms (with n=4,5,6,7) for the a-TiO_2 samples described in the text with 3000 and 162 atoms, respectively), calculated using a Ti-O bond length cutoff of 2.45 Å. The results are compared with a previous study analyzing the X-ray absorption near edge structure of ALD-prepared a-TiO_2.[S5]

	**Ti_4c**	**Ti_5c**	**Ti_6c**	**Ti_7c**
3000 atoms	4	42	51	3
162 atoms	4	32	60	5
XAFS (modeling)[S5]	0	50	47	3
XAFS (data fitting)[S5]	22	16	62	0

Table S2. The comparison of the average numbers of H_2O molecules either molecularly or dissociatively adsorbed on the TiO_2 side and $BiVO_4$ side of the interface in $BiVO_4$-2ML H_2O/a-TiO_2 and $BiVO_4$/H_2O-a-(Ti)TiO_2 after the FPMD simulations. The results are from **Figure S5 and Figure 7**, respectively.

**$BiVO_4$-2ML H_2O/a-TiO_2** (starting with 10 H_2O molecules initially adsorbed on $BiVO_4$)	**$BiVO_4$/H_2O-a-(Ti)TiO_2** (starting with 10 H_2O molecules initially adsorbed on Ti-rich a-TiO_2)
# of H_2O molecularly adsorbed on TiO_2: **3**	# of H_2O molecularly adsorbed on TiO_2: **6**
# of H_2O dissociatively adsorbed on TiO_2: **1**	# of H_2O dissociatively adsorbed on TiO_2: **3**
# of H_2O molecularly adsorbed on $BiVO_4$: **4**	# of H_2O molecularly adsorbed on $BiVO_4$: **2**
# of H_2O dissociatively adsorbed on $BiVO_4$: **0**	# of H_2O dissociatively adsorbed on $BiVO_4$: **1**

Table S3. Curve fitting results from **Figure 10a-b** where the O 1s peak was fitted with two peaks.*

	**Peak 1** (Lattice O)	**Peak 2** (Ti-OH)
E_b (eV)	530.0	532.1
FWHM (eV)	1.8	2.2
Weights in $BiVO_4/TiO_2$(TTIP)	0.826	0.174
Weights in $BiVO_4/TiO_2(H_2O)$	0.818	0.182

Table S4. Curve fitting results from **Figure 10c-d** where the O 1s peak was fitted with three peaks.*

	**Peak 1** (Lattice O)	**Peak 2** (Ti-OH)	**Peak 3** (Interfacial H_2O)
E_b (eV)	530.0	532.2	531.0
FWHM (eV)	1.7	2.1	1.9
Weights in $BiVO_4/TiO_2$(TTIP)	0.788	0.176	0.000
Weights in $BiVO_4/TiO_2(H_2O)$	0.824	0.160	0.052

*In order to obtain results in Tables S3 and S4, the O 1s XPS spectra were deconvoluted into individual spectral components with a Gaussian line shape, using a code based on the scipy.optimize.curve_fit package. Given that the two samples are chemically very similar, the fitting was constrained so that for each peak component, it maintains the same binding energy and linewidth between the two samples, although the relative spectral weight can change freely.

Table S5. R_{CT} and C_{IT} values of $BiVO_4/TiO_2$(TTIP) and $BiVO_4/TiO_2(H_2O)$ obtained from the EIS results shown in **Figure 11**. The procedure is explained in **Figure S10**.

		**$BiVO_4/TiO_2$(TTIP)**	**$BiVO_4/TiO_2(H_2O)$**
**R_{CT} (Ω)**	0.65 V	5.49×10^{3}	3.86×10^{3}
	0.70 V	1.58×10^{3}	1.05×10^{3}
**C_{IT} (F)**	0.65 V	1.16×10^{-4}	1.33×10^{-4}
	0.70 V	1.08×10^{-4}	1.04×10^{-4}

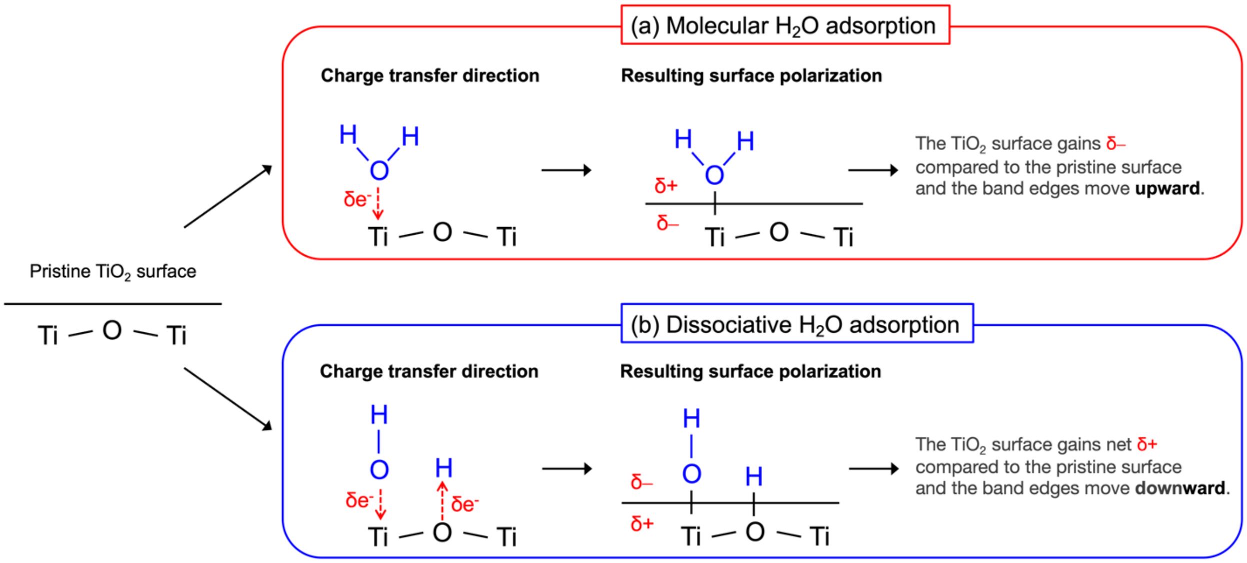

Scheme S1. Schematic representation of a single water molecule adsorbed on TiO_2 and the resulting change in surface polarization. When water is molecularly adsorbed (a), there is a charge transfer from the O atom in H_2O to the Ti in the TiO_2 surface. The resulting change in surface polarization leads to the shift of the band edges closer to the vacuum level.[S6] When water is dissociatively adsorbed (b), while there is a charge transfer from the O atom in OH to the Ti in the TiO_2 surface, there is also a charge transfer from the O atom in the TiO_2 surface to the H atom from H_2O such that the net charge transfer is from the TiO_2 surface to the dissociatively adsorbed water species. The resulting change in surface polarization leads to the shift of the band edges away from the vacuum level.[S6]

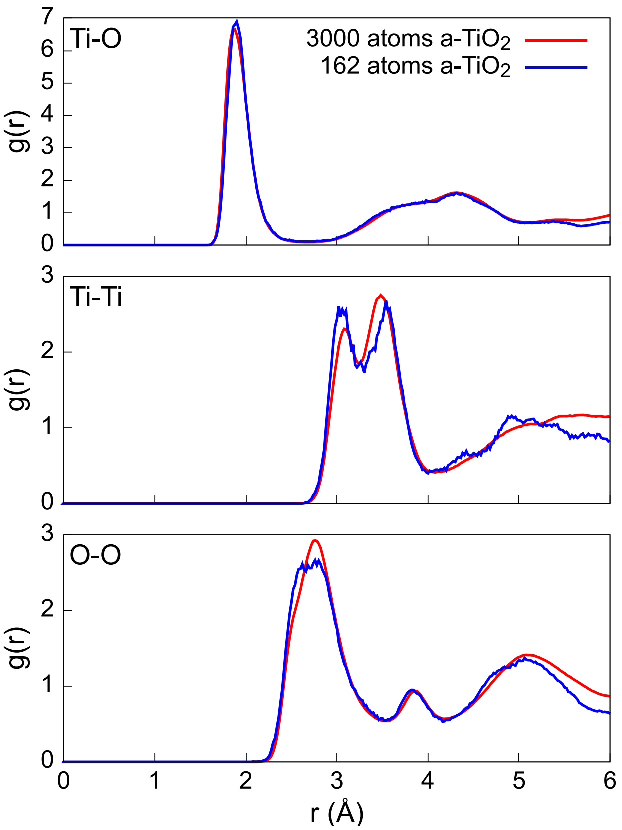

Figure S1. Radial distribution function (RDF) of bulk a-TiO_2 obtained from DPMD simulations. We show Ti-O, Ti-Ti, and O-O RDFs computed for cells with 162 and 3000 atoms.

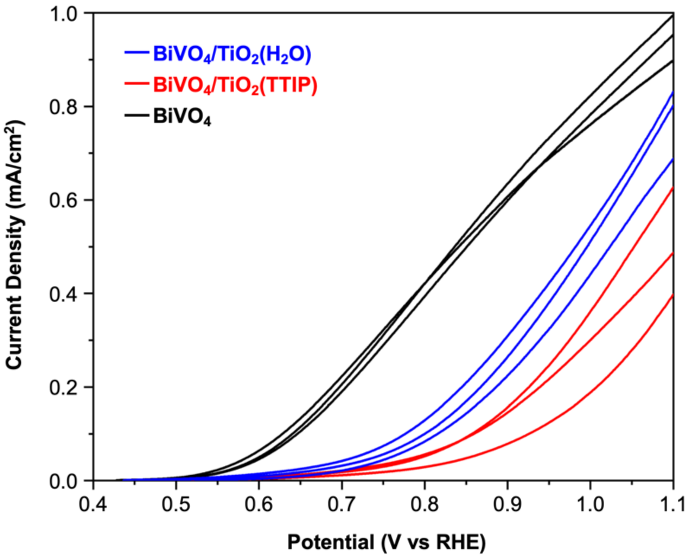

Figure S2. *J–V* plots for sulfite oxidation of $BiVO_4$ (black), $BiVO_4/TiO_2$(TTIP) (red), and $BiVO_4/TiO_2(H_2O)$ (blue) photoanodes. For each sample type, three different samples were used to obtain *J–V* plots, and they were used to prepare the averaged *J–V* plots with standard deviations shown in Figure 3. *J–V* plots were measured in 0.5 M borate buffer (pH 9.3) containing 0.4 M sodium sulfite under AM 1.5G illumination.

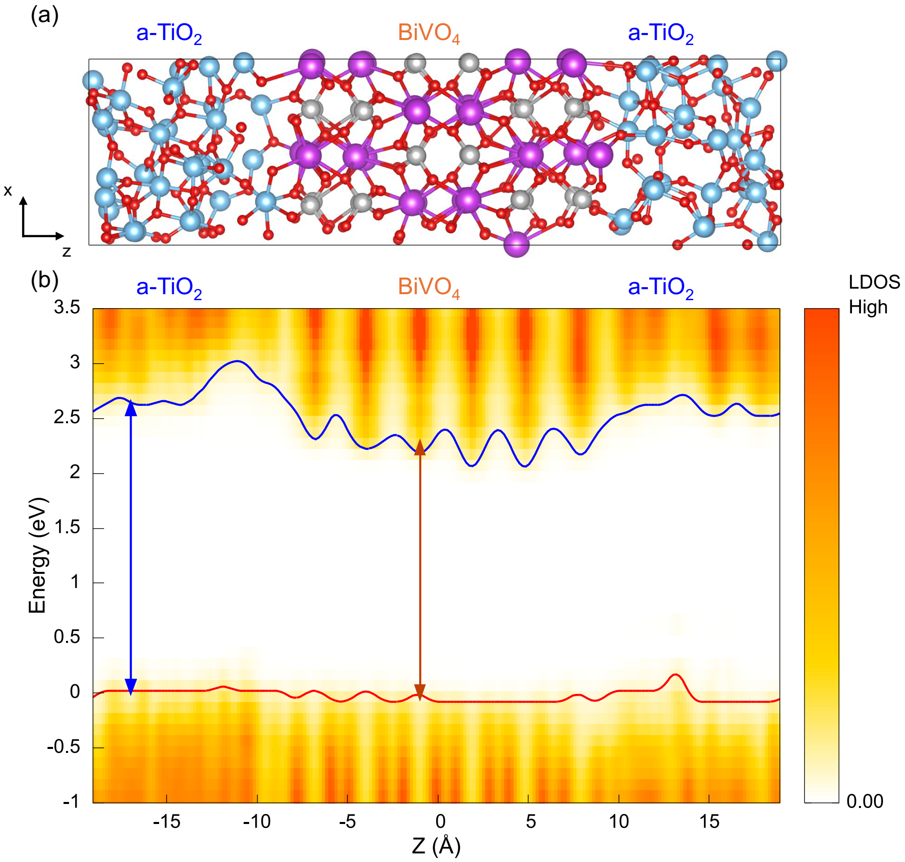

Figure S3. (a) Ball and stick representation of the $BiVO_4$/a-TiO_2 dry interface, where a-TiO_2 has a stoichiometric surface but a structure different from that of the model presented in the main text (color coding of spheres is the same as in Figure 4 in the main text). Specifically, we extracted an additional 162-atom configuration from the initial 3000 atom bulk a-TiO_2 generated by DPMD; we then optimized the extracted structure using the method described above and obtained an a-TiO_2 configuration with a stoichiometric surface. (b) Local density of states (LDOS) along the direction perpendicular to the interface (z direction). The solid blue and red lines represent the position in energy of the CBM and VBM in the slab, respectively. The blue and red arrows indicate the averaged energy gap in the a-TiO_2 and $BiVO_4$ regions of the slab, respectively.

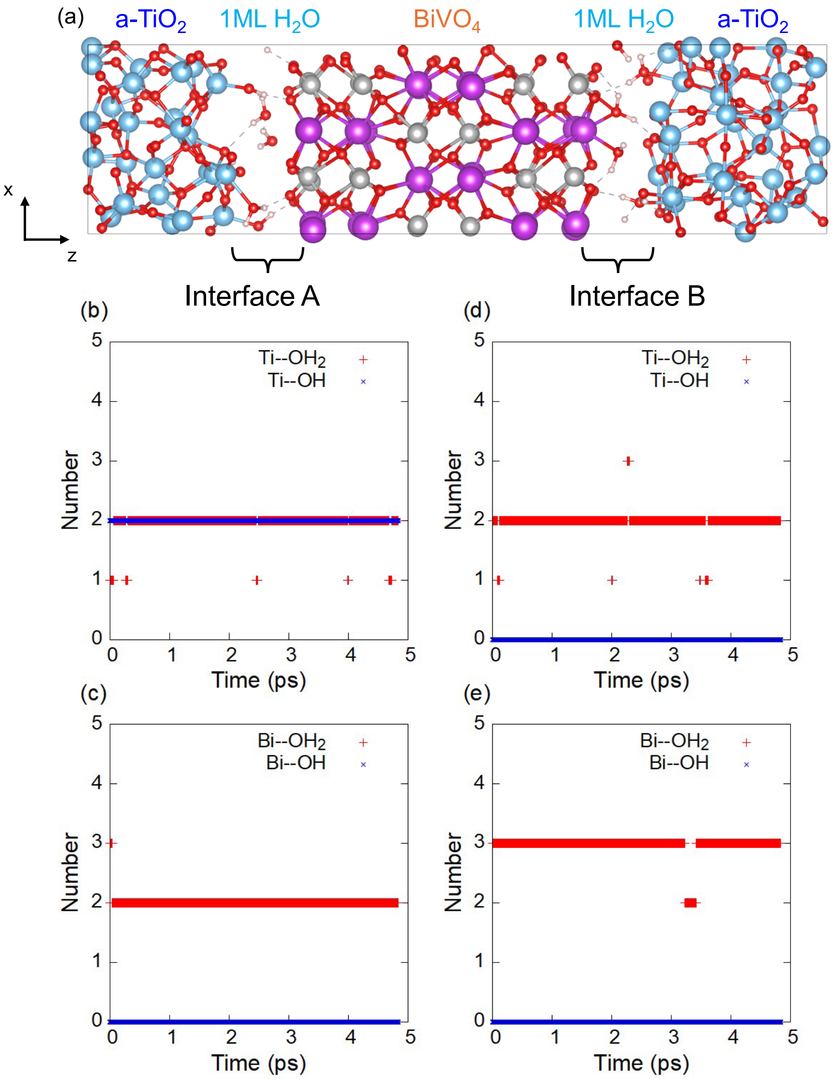

Figure S4. (a) A representative snapshot of the $BiVO_4$-1ML H_2O/a-TiO_2 interface (same color coding of spheres as Figure 4a) obtained in our 300 K FPMD simulation (see main text). Note that in our slab two $BiVO_4$/a-TiO_2 interfaces (named A and B) are shown because our slab model is periodically repeated. As these two interfaces do not necessarily have identical atomic structures since two a-TiO_2 surfaces are not identical, we show the numbers of water molecules molecularly and dissociatively adsorbed on (b,d) Ti atoms on the a-TiO_2 side and (c,e) Bi atoms on the $BiVO_4$ side as a function of the simulated time, for interfaces A (b,c) and B (d,e) separately. M-OH_2 where M is Bi or Ti represents molecularly adsorbed H_2O, and M-OH represents dissociatively adsorbed H_2O. Note that one dissociatively adsorbed H_2O generates two M-OH bonds, one with OH from water adsorbing on M (M-OH) and the other with H from water adsorbing on O in the solid lattice connected to M (MO-H), which are both counted as M-OH in (d-e). The cutoff radii used to define Bi—O and Ti—O bonds are 3.20 Å and 2.45 Å, respectively. We find that some of the water molecules initially adsorbed on the $BiVO_4$ surface are desorbed from $BiVO_4$ and adsorbed on a-TiO_2 after the interface is formed. These results suggest that water molecules may be preferentially adsorbed on a-TiO_2.

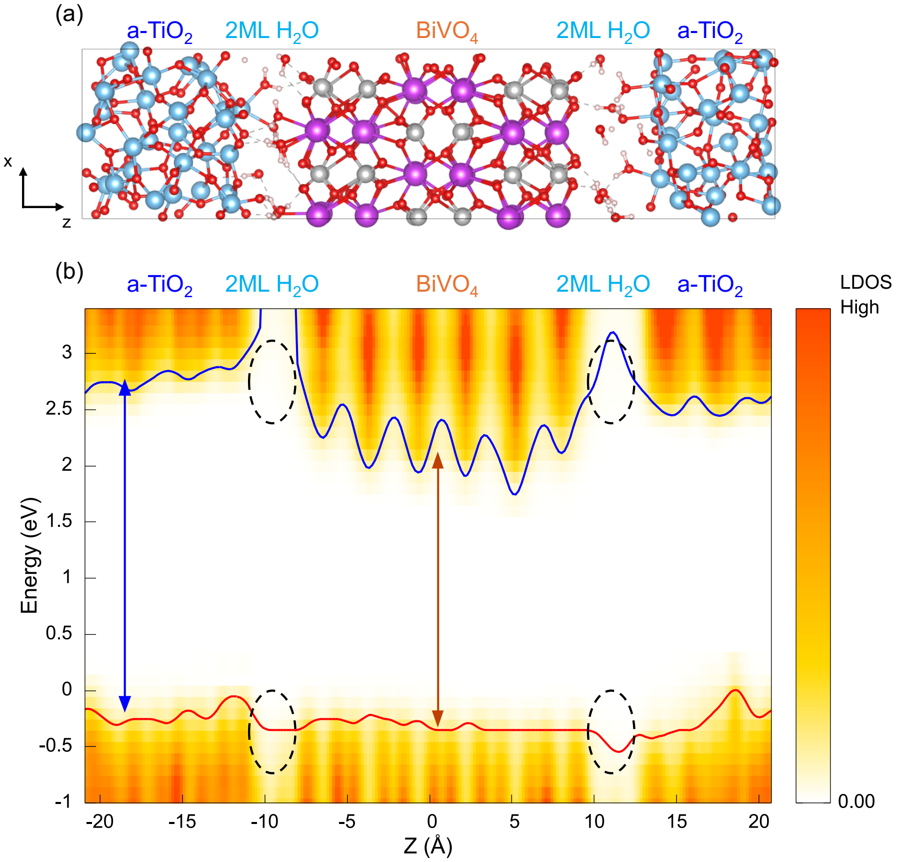

Figure S5. (a) Ball and stick representation of the $BiVO_4$-2ML H_2O/a-TiO_2 interface (same color coding of spheres as Figure 4a). (b) Local density of states (LDOS) along the direction perpendicular to the interface (z direction). The solid blue and red lines represent the position in energy of the CBM and VBM in the slab, respectively. The blue and red arrows indicate the averaged energy gap in the a-TiO_2 and $BiVO_4$ regions of the slab, respectively. The black dashed circles indicate the $BiVO_4$/a-TiO_2 interfacial regions where the presence of water molecules led to a depletion of electronic states.

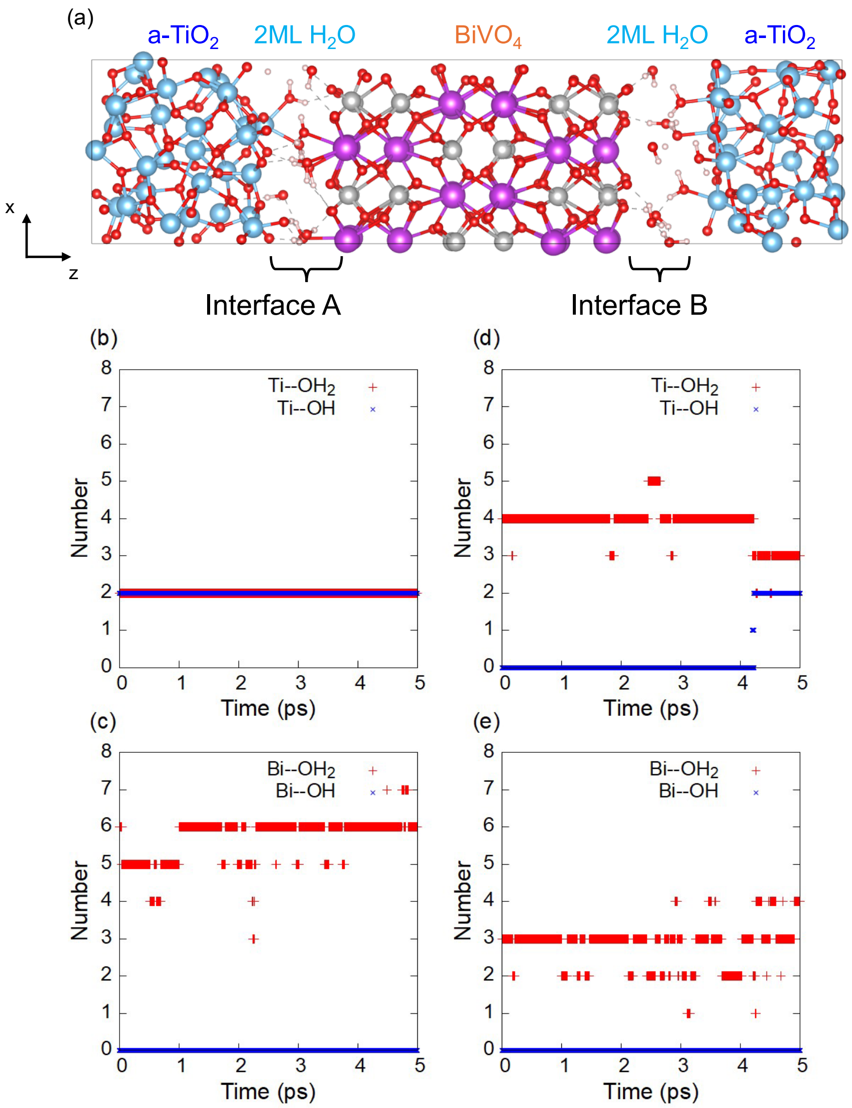

Figure S6. (a) A representative snapshot of the $BiVO_4$-2ML H_2O/a-TiO_2 interface (same color coding of spheres as Figure 4a) obtained in our 300 K FPMD simulation (see main text). Note that in our slab two $BiVO_4$/a-TiO_2 interfaces (named A and B) are shown because our slab model is periodically repeated. As these two interfaces do not necessarily have identical atomic structures since two a-TiO_2 surfaces are not identical, we show the numbers of water molecules molecularly and dissociatively adsorbed on (b,d) Ti atoms on the a-TiO_2 side and (c,e) Bi atoms on the $BiVO_4$ side as a function of the simulated time, for interfaces A (b,c) and B (d,e) separately. M-OH_2 where M is Bi or Ti represents molecularly adsorbed H_2O, and M-OH represents dissociatively adsorbed H_2O. Note that one dissociatively adsorbed H_2O generates two M-OH bonds, one with OH from water adsorbing on M (M-OH) and the other with H from water adsorbing on O in the solid lattice connected to M (MO-H), which are both counted as M-OH in (d-e). The cutoff radii used to define Bi—O and Ti—O bonds are 3.20 Å and 2.45 Å, respectively.

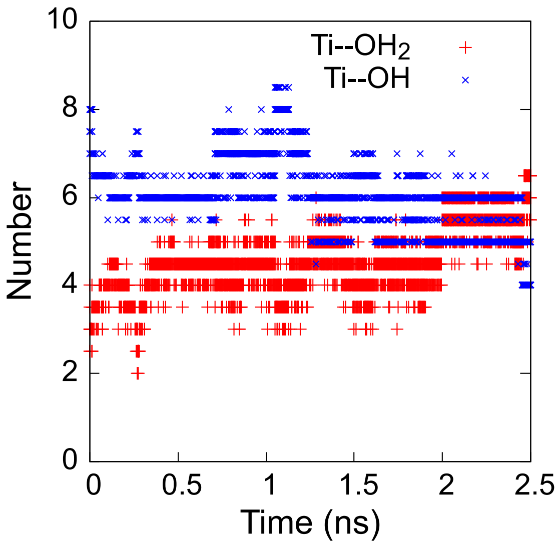

Figure S7. The number of Ti-OH_2 and Ti-OH bonds formed by water adsorption as a function of the simulation time for the Ti-rich a-TiO_2 surface. The simulations were carried out with the DPMD potential. Ti-OH_2 bonds are formed by water adsorbing as a molecule and Ti-OH bonds are formed by the water dissociative adsorption. The cutoff radius used to define the Ti-O bond is 2.45 Å.

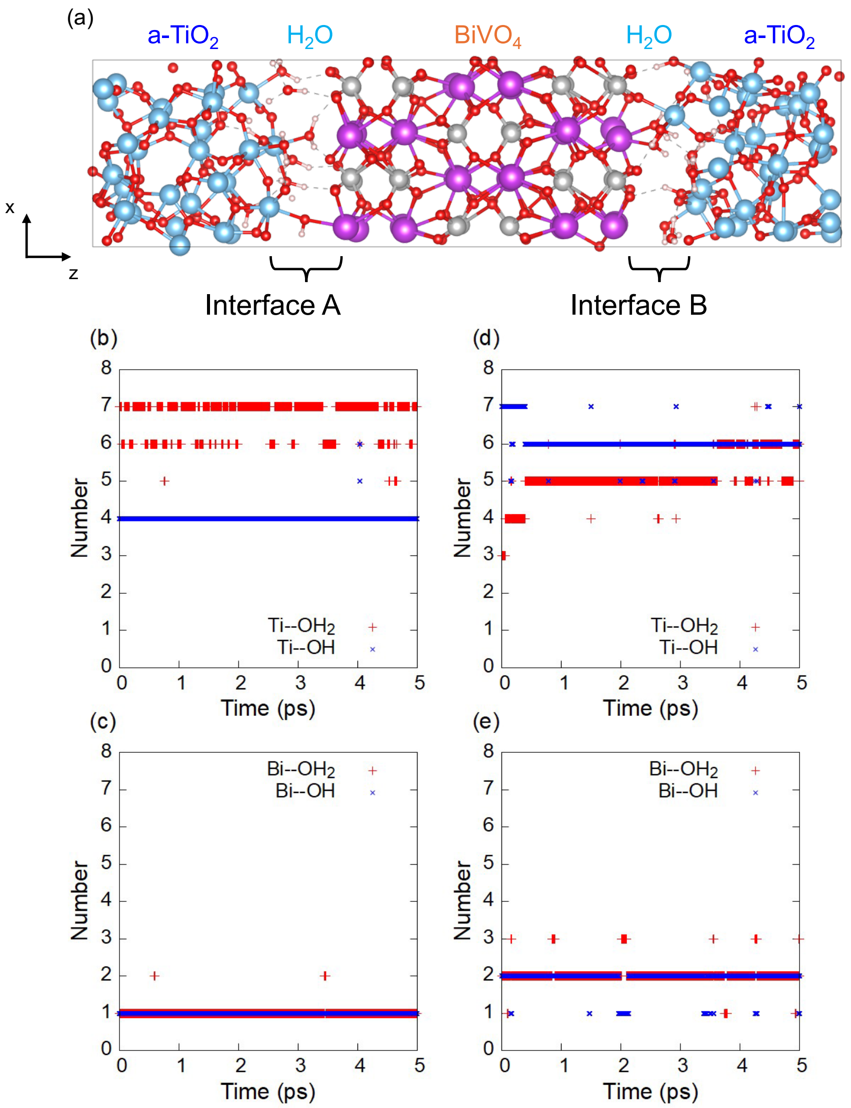

Figure S8. (a) A representative snapshot of the $BiVO_4$/H_2O-a-(Ti)TiO_2 interface (same color coding of spheres as Figure 4a) obtained in our 300 K FPMD simulation (see main text). Note that in our slab two $BiVO_4$/a-TiO_2 interfaces (named A and B) are shown because our slab model is periodically repeated. As these two interfaces do not necessarily have identical atomic structures since two a-TiO_2 surfaces are not identical, we show the numbers of water molecules molecularly and dissociatively adsorbed on (b,d) Ti atoms on the a-TiO_2 side and (c,e) Bi atoms on the $BiVO_4$ side as a function of the simulated time, for interfaces A (b,c) and B (d,e) separately. M-OH_2 where M is Bi or Ti represents molecularly adsorbed H_2O, and M-OH represents dissociatively adsorbed H_2O. Note that one dissociatively adsorbed H_2O generates two M-OH bonds, one with OH from water adsorbing on M (M-OH) and the other with H from water adsorbing on O in the solid lattice connected to M (MO-H), which are both counted as M-OH in (d-e). The cutoff radii used to define Bi—O and Ti—O bonds are 3.20 Å and 2.45 Å, respectively. Note that the number of dissociatively adsorbed H_2O on TiO_2 is higher at Interface B than at Interface A, and this results in a higher density of states from H_2O near the VBM of TiO_2 in the interfacial region as shown in Figure 7b.

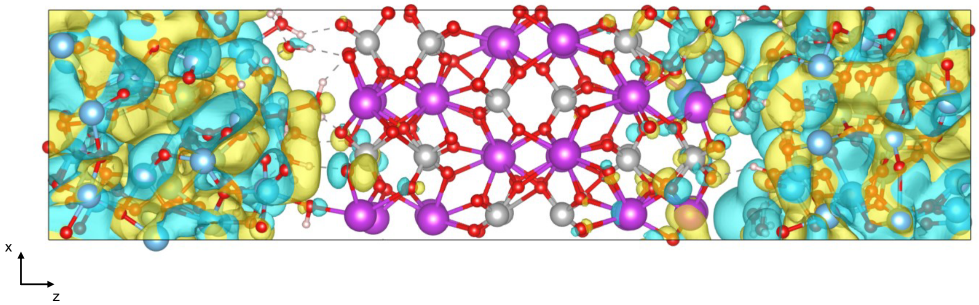

Figure S9. Iso-surface of the sum of wavefunctions close to the VBM [~0.3 eV below the VBM] showing the contribution of H_2O orbitals to near the VBM of the TiO_2 side of the $BiVO_4/H_2O$-a-(Ti)TiO_2 interface (same color coding of spheres as Figure 4a). Note that the interface structure of a-TiO_2 is not the same on both sides of the slabs, hence the electronic structure of water at the interfaces is slightly different in two interfaces. The iso-surface levels were set at $0.5a_0^{-3/2}$ (yellow) and $-0.5a_0^{-3/2}$ (blue), where a_0 is the Bohr radius.

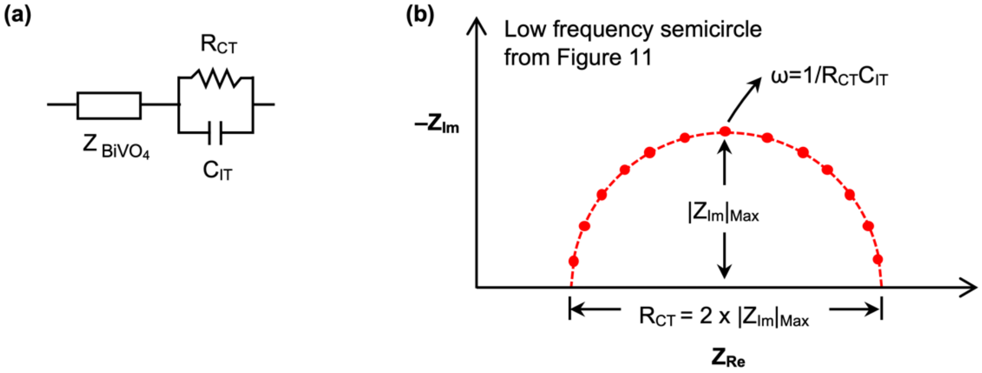

Figure S10. (a) An equivalent circuit used to obtain R_{CT} and C_{IT} values for $BiVO_4/TiO_2$(TTIP) and $BiVO_4/TiO_2(H_2O)$ originating from the addition of TiO_2(TTIP) and $TiO_2(H_2O)$. Z_{BiVO4} is the impedance originating from the common components of the $BiVO_4/TiO_2$(TTIP) and $BiVO_4/TiO_2(H_2O)$ samples (e.g., $BiVO_4$ and the $BiVO_4$/ITO back contact). (b) Schematic illustration explaining how to obtain R_{CT} and C_{IT} in **Table S5** from the low frequency semicircle in **Figure 11**.[S7] First, the radius of the semicircle was obtained from the maximal value of the imaginary part of impedance ($|Z_{Im}|_{Max}$). By doubling this value, the diameter of the semicircle is obtained, which is the value of R_{CT}. Then, C_{IT} was obtained using the following equation where ω is the angular frequency (ω=2πf, where f is the frequency) that yielded $|Z_{Im}|_{Max}$.

$$\omega \text{ at } |Z_{Im}|_{Max} = 1/R_{CT}C_{IT}$$

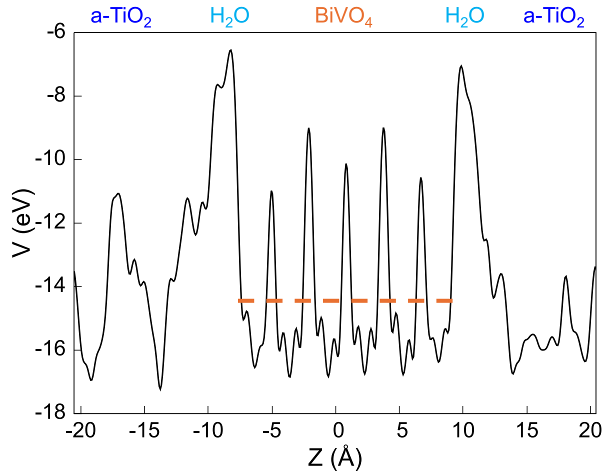

Figure S11. Electrostatic potential of the $BiVO_4/H_2O$-a-(Ti)TiO_2 interface structure projected along the direction (z) perpendicular to the interface. The orange dashed line indicates the averaged electrostatic potential along the z direction in the $BiVO_4$ region, which is flat. This is indicative of no significant total dipole moment present across the entire simulation cell.

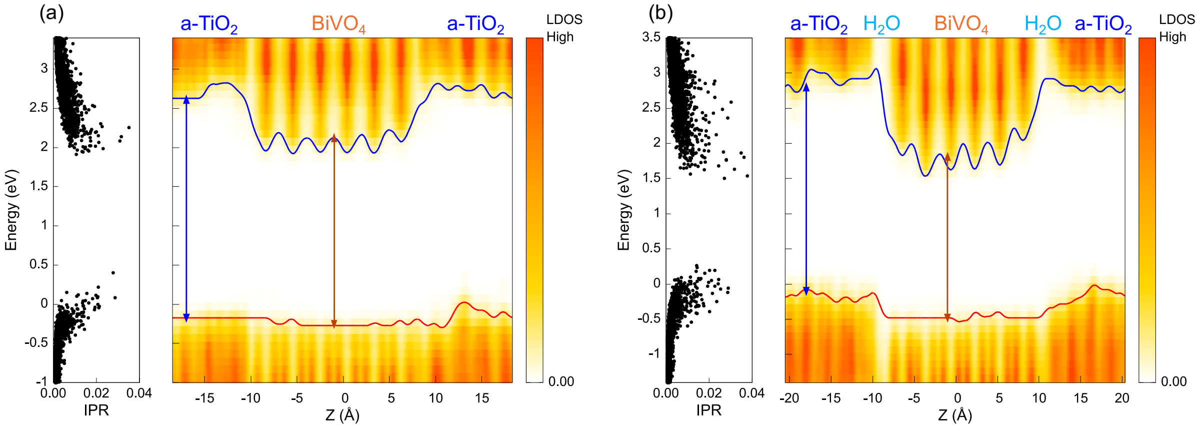

Figure S12. Local density of states (LDOS) along the direction perpendicular to the interface (z direction), right panel, and the inverse participation ratio (IPR) of the Kohn-Sham states as a function of energy, left panel, of (a) $BiVO_4$/a-TiO_2 interface and (b) $BiVO_4/H_2O$-a-(Ti)TiO_2 interface. The IPR of the orbital Ψ_i is defined as $\int|\Psi_i|^4 d^3r/(\int|\Psi_i|^2 d^3r)^2$. The higher the IPR value, the more an orbital is localized. In both cases, localized levels are present at 0.2-0.3 eV above the VBM, and no significant difference is observed in terms of the position and the density of the localized levels between the two samples. This result suggests that the regular and Ti-rich a-TiO_2 surfaces contain comparable levels of defects like Ti^{3+} at the interface.

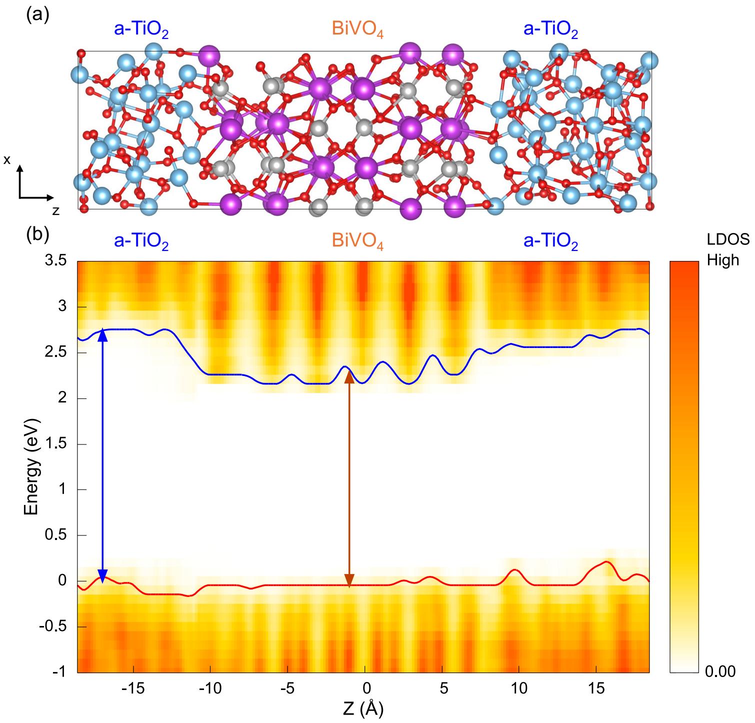

Figure S13. (a) Ball and stick structure of a *dry* $BiVO_4$/a-(Ti)TiO_2 interface, where a Ti-rich a-TiO_2 surface is interfaced with $BiVO_4$ without interfacial water molecules (color coding of spheres is the same as in Figure 4 in the main text). (b) LDOS along the direction perpendicular to the interface (z direction). The solid blue and red lines represent the position in energy of the CBM and VBM in the slab, respectively. The blue and red arrows indicate the averaged energy gap in the a-TiO_2 and $BiVO_4$ regions of the slab, respectively. The LDOS of the *dry* $BiVO_4$/a-(Ti)TiO_2 interface closely resembles that of the $BiVO_4$/a-TiO_2 model shown in **Figure 5b** in the main text; in particular, the valence band offset between $BiVO_4$ and a-TiO_2 is almost identical. This comparison confirms that the favorable interfacial band alignment of $BiVO_4$/H_2O-a-(Ti)TiO_2 is from the presence of interfacial H_2O interacting with the Ti-rich a-TiO_2 surface and not from the Ti-rich a-TiO_2 surface.

References

(S1) Calegari Andrade, M. F.; Selloni, A. Structure of Disordered TiO_2 Phases from *Ab Initio* Based Deep Neural Network Simulations. *Phys. Rev. Mater.* **2020**, *4*, 113803.

(S2) Ding, Z.; Selloni, A. Modeling the Aqueous Interface of Amorphous TiO_2 Using Deep Potential Molecular Dynamics. *J. Chem. Phys.* **2023**, *159*, 024706.

(S3) Plimpton, S. Fast Parallel Algorithms for Short-Range Molecular Dynamics. *J. Comput. Phys.* **1995**, 117, 1–19.

(S4) Wang, H.; Zhang, L.; Han, J.; E, W. DeePMD-Kit: A Deep Learning Package for Many-Body Potential Energy Representation and Molecular Dynamics. *Comput. Phys. Commun.* **2018**, 228, 178–184.

(S5) Yan, D.; Topsakal. M.; Selcuk, S.; Lyons, J. L.; Zhang, W.; Wu, Q.; Waluyo, I.; Stavitski, E.; Attenkofer, K.; Yoo, S.; Hybertsen, M. S.; Lu, D.; Stacchiola, D. J.; Liu, M. Ultrathin Amorphous Titania on Nanowires: Optimization of Conformal Growth and Elucidation of Atomic-Scale Motifs. *Nano Lett.* **2019**, *19*, 3457–3463.

(S6) Han, F.; Zhou, Z.; Huang, Z.; Li, M.; Guo, L. Effect of Water Adsorption on the Interfacial Structure and Band Edge Alignment of Anatase TiO_2(001)/Water by First-Principles Molecular Dynamics. *J. Phys. Chem. C* **2018**, 122, 47, 26965–26973.

(S7) Bard, A. J.; Faulkner, L. R. Electrochemical Impedance Spectroscopy and ac Voltammetry. Electrochemical Methods: Fundamentals and Applications, 3nd ed.; Wiley, 2022; pp 443−488.
